# Comparative effectiveness research with average hazard for censored time-to-event outcomes: simulation study and application to observational data

**DOI:** 10.1186/s12874-025-02741-9

**Published:** 2025-12-26

**Authors:** Hong Xiong, Jean Connors, Deb Schrag, Hajime Uno

**Affiliations:** 1 Department of Biostatistics, Harvard T. H. Chan School of Public Health, Boston, Massachusetts 02115 USA; 2 Department of Hematologic Oncology, Dana Farber Cancer Institute, Boston, Massachusetts 02215 USA; 3 Department of Medicine, Memorial Sloan Kettering Cancer Center, New York, New York 10065 USA; 4 Department of Data Science, Dana Farber Cancer Institute, Boston, Massachusetts 02215 USA

**Keywords:** Adjusted survival curves, Causal inference, Cumulative incidence probability, Person-time incidence rate, Restricted mean survival time

## Abstract

**Background:**

The average hazard is a summary measure of event time distributions with a given time window, $[0,\tau ],$ and allows intuitive interpretation as an average person-time incidence rate over the time window. This metric is calculated as the ratio of the cumulative incidence probability at $\tau$ to the restricted mean survival time at $\tau$ and can be estimated through non-parametric methods and thus robust. While previously proposed for randomized trials, its use in comparative effectiveness research remains underexplored.

**Methods:**

We evaluate inference procedures for the difference and ratio of average hazards from two comparative groups, using six common confounding adjustment methods for survival functions, including direct standardization, inverse probability of treatment weighting (IPTW), propensity score matching, empirical likelihood, and augmented IPTW (AIPTW). Extensive simulation studies under varying model specification are conducted to assess bias, variance, coverage probability, and width of confidence interval. We apply the method to data from the preference cohort in the CANVAS study.

**Results:**

All adjustment methods achieved satisfactory performance; AIPTW was notably robust under partial model misspecification.

**Conclusions:**

Using difference in average hazards and ratio of average hazards as estimands, when combined with common confounding adjustment methods, is feasible and reliable for comparative effectiveness research. The average hazard-based analysis provides a practical alternative to the traditional hazard ratio approach for quantifying the magnitude of the intervention effect on survival outcomes.

**Supplementary Information:**

The online version contains supplementary material available at 10.1186/s12874-025-02741-9.

## Introduction

For decades, the hazard ratio (HR), derived from Cox’s proportional hazards (PH) model [1], has been the predominant metric for quantifying treatment effects on time-to-event outcomes in clinical research. While Cox’s HR benefits from desirable statistical properties and an elegant theoretical foundation, its interpretation becomes problematic when the PH assumption is violated [2–6]. Under non-proportional hazards, the estimated HR is influenced by the study-specific censoring distribution, which compromises its generalizability to other settings or populations [7–10]. To address this limitation, the average hazard ratio (aHR) was proposed by Kalbfleisch and Prentice [7] and further developed and discussed by other authors [11–13]. The aHR is defined as a weighted average of the time-specific HRs. Unlike Cox’s HR, the aHR does not rely on the PH assumption and can be consistently estimated under general censoring mechanisms.

However, an important limitation remains from the perspective of clinical interpretation: neither Cox’s HR nor aHR is designed to estimate group-specific absolute hazards, as both focus solely on the relative contrast between groups. From a statistical standpoint, it is generally more efficient to estimate the HR or aHR directly rather than to first estimate the absolute hazards in each group and then compute their ratio. Nevertheless, the lack of group-specific absolute hazard estimates constrains the interpretability of these relative measures. For example, an HR or aHR of 0.97 numerically suggests a small treatment effect. Yet, its clinical interpretation depends on the absolute hazard in the control group: if the baseline hazard in the control group is low, a 3% reduction in hazard may be a negligible improvement by the treatment; if it is high, the same relative reduction could indicate a meaningful clinical benefit. Conversely, even a seemingly large relative effect (e.g., HR or aHR = 0.5) may lack clinical significance if the baseline hazard in the control group is very low.

Having access to group-specific absolute hazard estimates would enable simultaneous presentation of treatment effects in both absolute and relative terms, as recommended by the Annals of Internal Medicine’s statistical guidance for survival outcomes [14]. A similar recommendation exists for binary outcomes in the CONSORT 2025 guidelines [15]. Without such group-specific summary measures, yielding the between-group contrast measures such as ratios or absolute differences, it becomes difficult to assess treatment benefit from a clinical perspective, potentially limiting the interpretability of study findings.

Given these limitations, alternative approaches have been proposed to quantify between-group differences using summary measures of the event time distribution, such as median survival, cumulative incidence probability, and restricted mean survival time (RMST). Notably, these summary measures can be estimated non-parametrically without imposing strong modeling assumptions and are thus robust [5]. The summary measures of the event time distributions from the treatment group and control group easily allow for the expression of the treatment effect magnitude in both absolute difference and relative terms. This enhances the interpretation of the treatment effect magnitude, as suggested by the aforementioned guidelines [14, 15].

While the RMST-based approach in this class is gaining more attention [3–6] and is beginning to be used in practice [16–19], the average hazard with survival weight (AH) has emerged more recently as another promising approach in this class [20–22]. The AH (or generalized hazard [23, 24]) is defined as

1 $$\begin{aligned} \eta (\tau ) = \frac{\int _{0}^{\tau } h(u)S(u)du}{\int _{0}^{\tau } S(u)du} = \frac{E\{I(T\le \tau )\}}{E\{ T \wedge \tau \}}, \end{aligned}$$

where $\tau$ is a truncation time point, *h*(*u*) and *S*(*u*) are the hazard function and survival function for the event time *T*, respectively, *I*(*A*) is an indicator function for the event *A*, and $x \wedge y$ denotes $\min (x,y).$ The AH is closely related to the person-time incidence rate (IR) commonly defined as *‘the number of observed events divided by the total observation time’* [25] that is,

2 $$\begin{aligned} IR = \frac{\sum \nolimits _{i=1}^{n}I(T_i \le C_i)}{\sum \nolimits _{i=1}^{n}( T_i \wedge C_i )}, \end{aligned}$$

where *n* is the sample size of the analysis population, and $\{ T_i \}$ and $\{ C_i \}$ for $i=1 \ldots , n$ are the independent copies from the event time *T* and censoring time *C*, respectively. As $n \rightarrow \infty ,$ this converges to

3 $$\begin{aligned} \frac{E\{ I(T \le C) \} }{E\{ T \wedge C \} }. \end{aligned}$$

Note that this quantity depends on the censoring time distribution, except in special cases where the event time *T* follows an exponential distribution. On the other hand, (1) does not involve *C* but a fixed time point $\tau .$ In contrast to the conventional person-time incidence rate, (2) or (3), the AH provides an average person-time incidence rate over a specified time window, removing the effect of the nuisance censoring time distribution. It should be noted that, as with the conventional IR, the unit of measurement for AH is “events per person-time,” which allows for unambiguous interpretation. For example, if the estimated AH with truncation time $\tau =24$ months is 0.4, this indicates that we would observe 0.4 events per person-month on average during the 24-month period in the absence of censored observations.

For a comparison between Group 0 and 1, difference in AH (DAH) and ratio of AH (RAH) can be defined as

4 $$\begin{aligned} \eta _1(\tau )-\eta _0(\tau ) = \frac{1-S_1(\tau )}{\int _{0}^{\tau } S_1(u)du} - \frac{1-S_0(\tau )}{\int _{0}^{\tau } S_0(u)du}, \end{aligned}$$

and

5 $$\begin{aligned} \eta _1(\tau )/\eta _0(\tau ) = \left\{ \frac{1-S_1(\tau )}{1-S_0(\tau )} \right\} \left\{ \frac{\int _{0}^{\tau } S_0(u)du}{\int _{0}^{\tau } S_1(u)du}\right\} , \end{aligned}$$

respectively, where $S_k(t)$ is the survival function for the group *k*. It is important to note that the RAH (i.e., ratio of average hazards) (5) is a fundamentally different quantity from the *average hazard ratio* (aHR) [7] introduced earlier, despite the similarity in naming. A non-parametric approach for the inference of DAH and RAH is to plug in the Kaplan-Meier (KM) estimates for $S_1(t)$ and $S_0(t)$ in equations (4) and (5). This approach has already been investigated under randomized trial settings by Uno and Horiguchi [20]. An R package (survAH) [26] is also available for implementation of the KM-based inference.

It is important to note that there are several appealing features of using the AH, compared with the survival function or RMST. First, as shown in Equation (1), the AH is the ratio of cumulative incidence probability at $\tau$ to RMST with truncation time $\tau .$ Therefore, AH is generally less sensitive to the choice of $\tau$ than cumulative incidence probability or RMST, offering a more robust summary across different time horizons. A result of numerical investigation with this regard is seen in Uno et al. [21]. Second, unlike cumulative incidence probability or RMST, AH provides a hazard-based summary that connects naturally to the widely used Cox’s hazard-based analysis, thereby offering continuity with the familiar concept of hazard ratio. In terms of power, power advantages depend on the underlying patterns of difference between two survival curves. Specifically, RMST tends to be more powerful when treatment differences occur early, whereas cumulative incidence probability is more powerful when differences occur late; AH, as a combination of the two, typically yields intermediate power across a range of scenarios [20].

Currently, the application of the AH-based approach to general comparative effectiveness research (CER), where interventions are not allocated through randomization, has yet to be well investigated. In general within CER, the KM-based approach previously outlined for calculating DAH and RAH falls short in adequately adjusting for variations in case-mix between groups and can result in biased estimates of differences between groups. However, it is evident from equations (4) and (5) that DAH and RAH are derived as functions of the survival functions of the respective groups. This suggests that the AH approach can be seamlessly integrated into CER by combining it with existing methods developed to derive *adjusted* survival functions [27–34]. Yet, the statistical performance of this combined approach remains underexplored for AH.

This paper seeks to address the existing gap by merging established techniques for deriving adjusted survival functions with the AH-based approach in CER. Through extensive numerical studies, we evaluated the statistical performance of these integrated methods. We explored the practical applicability of these approaches for real-world CER studies, supplemented by an illustrative example using real data from an effectiveness study evaluating direct oral anticoagulants (DOAC) compared with low-molecular-weight heparin (LMWH) for the prevention of recurrent venous thrombosis (VTE) events in cancer patients who experienced an initial VTE event [35].

## Methods and materials

### Overview

The objective of our numerical studies was to systematically evaluate the performance of various statistical methods for adjusting survival curves under different experimental settings when performing statistical inference regarding the treatment effects on survival time outcomes using AH as the summary measure of the survival time distribution.

For each experimental setting, survival data were generated and analyzed by various confounding adjustment methods with details described in the next subsection. We employed several specialized R packages, including the *survival*, *survAH*, *riskRegression*, *pammtools*, *Matching*, *geepack*, and *adjustedCurves* packages.

For each analytical method, the adjusted survival curves $\hat{S}_1(t)$ and $\hat{S}_0(t)$ were computed for the treatment (Group 1) and control (Group 0) groups, respectively. The adjusted AH’s were calculated as $\hat{\eta }_1(\tau ) = \left\{ 1-\hat{S}_1(\tau )\right\} /{\int _{0}^{\tau } \hat{S}_1(u)du}$ and $\hat{\eta }_0(\tau ) = \left\{ 1-\hat{S}_0(\tau )\right\} /{\int _{0}^{\tau } \hat{S}_0(u)du},$ respectively. The treatment effect parameters of interest, DAH and RAH, were then computed by $\hat{\eta }_1(\tau ) - \hat{\eta }_0(\tau )$ and $\hat{\eta }_1(\tau ) / \hat{\eta }_0(\tau ),$ respectively. Corresponding 95% confidence intervals were also computed using a standard error that was derived by bootstrapping with 300 resamples. We chose bootstrapping for our numerical studies because not all included confounding-adjustment methods had an available analytical variance formula. Adopting bootstrap confidence intervals for all methods, regardless of the availability of an analytic variance formula or software, allows for a consistent resampling-based approach to comparing the performance of the adjustment methods.

This process from data generation to analysis was repeated 2000 times. The final step of our simulation studies involved collating and summarizing the results across all iterations, evaluating each method through statistical performance metrics regarding inference of DAH, RAH, ${\eta }_0(\tau ),$ and ${\eta }_1(\tau ).$

### Confounding adjustment methods outline

The following six confounding adjustment methods were selected based on Denz et al. [36] and included in our simulation study: 1) Direct Standardization, 2) Inverse Probability of Treatment Weighted Kaplan-Meier Estimates, 3) Inverse Probability of Treatment Weighted Survival via Cumulative Hazard, 4) Propensity Score Matching, 5) Empirical Likelihood Estimation, and 6) Augmented Inverse Probability of Treatment Weighting. It is also theoretically possible to combine these approaches with the pseudo-value approach used for adjustment of censored observations [37]. However, due to our focus on main approaches for confounding adjustment, we excluded methods that combine these with the pseudo-value approach. Additionally, the standard Kaplan-Meier approach was included as a reference to demonstrate the effect of the absence of confounding adjustment. A brief introduction to each method is given below, and more details with mathematical expressions along with assumptions on censoring are provided in Supplementary Appendix A.

#### 1) Direct Standardization

The Direct Standardization (DS) method, or “G-Formula”, estimates adjusted survival curves via the Cox regression model [27, 28, 38]. This method fits the data to a Cox regression model, including the treatment group indicators and confounding factors. Based on the fitted model, the predicted survival curves are derived for all subjects in the dataset, assigning them to Group 1. The adjusted survival curve for Group 1 is then given by the average of these predicted survival probabilities. This is then repeated in the same manner to give the adjusted survival curve for Group 0. The validity of this approach relies on the adequacy of the specified model to predict the time-to-event outcome.

#### 2) Inverse Probability of Treatment Weighted Kaplan-Meier Estimates

In the Inverse Probability of Treatment Weighted Kaplan-Meier Estimates (IPTW KM) approach, the Kaplan-Meier estimator is applied to samples that are weighted using the Inverse Probability of Treatment Weighting (IPTW) method to yield adjusted survival curves. The IPTW methodology is designed to address confounding by modeling the mechanism of treatment assignment [39]. Typically, logistic regression is employed to calculate propensity scores for each individual across different levels of treatment [40]. Subsequently, individuals are assigned weights based on these estimated propensity scores. The Kaplan-Meier estimator is then used on these weighted samples to generate adjusted survival curves [30]. Assuming accurate modeling and estimation of weights, this method effectively mitigates confounding, leading to consistent estimates.

#### 3) Inverse Probability of Treatment Weighted Survival via Cumulative Hazard

The Inverse Probability of Treatment Weighted Survival via Cumulative Hazard (IPTW CH) approach was introduced by Cole and Hernán [29]. It utilizes a stratified Cox regression model in conjunction with IPTW. In the Cox model, confounding factors are not included; instead, the model includes only the treatment group indicator as a stratification factor and utilizes IPTW for individual weighting. Statistical software, such as the *coxph* function in R and *PROC PHREG* in SAS, is used to calculate the adjusted cumulative hazard function for each treatment group. The adjusted survival function is subsequently obtained by exponentiating the negative of this adjusted cumulative hazard function.

#### 4) Propensity Score Matching

Propensity Score Matching (Matching) represents an alternative, well-established method to control for confounding by employing the application of propensity scores [40]. This approach differs by using propensity scores for individual weighting. Instead, it involves the creation of a new dataset, composed of individuals who exhibit similar estimated propensity scores. This methodology aims to achieve a balanced distribution of confounding factors across treatment groups. Specifically, it adopts a bidirectional matching approach in which each treated patient is matched to a control patient and, conversely, each control patient is matched to a treated patient. This construction preserves representation from both treatment groups, ensuring that the resulting matched sample corresponds to the overall study population. Accordingly, the estimand targeted by this method is the average treatment effect in the overall population, rather than an effect limited to one subgroup. Subsequently, the Kaplan-Meier estimator is applied to these matched samples, enabling the derivation of the adjusted survival curve for each group. Assuming correct specification of the propensity score model and the efficacy of the matching algorithm, the resultant survival curves are expected to be consistent [31].

#### 5) Empirical Likelihood Estimation

Empirical Likelihood Estimation (EL) represents a non-parametric approach grounded in likelihood estimation [32]. By maximizing a constrained likelihood function, the EL methodology aims to align moments of covariates between treatment groups, thus achieving similarity in distributional characteristics and removing bias. Notably, given the absence of a specified outcome model and treatment assignment model, the EL approach exhibits enhanced robustness to potential model misspecifications compared to methods necessitating a model, such as IPTW.

#### 6) Augmented Inverse Probability of Treatment Weighting

Augmented Inverse Probability Weighting (AIPTW) approach marks a notable advancement in statistical methodologies for causal inference. Initially proposed by Robins and Rotnitzky [33], it has been further refined by various researchers in different contexts [34, 41–43]. This method distinctively integrates both the outcome and treatment models, leveraging the strengths of each to enhance the estimation process. Specifically, AIPTW utilizes the outcome model to enhance the IPTW estimate based on the treatment model, with the goal of improving its efficiency. This approach ensures asymptotic unbiasedness, provided that at least one of the models — either the outcome model or the treatment model — is correctly specified. This grants AIPTW its notable doubly robust property. This feature is a significant strength of AIPTW, offering a robust layer of protection in the face of potential inaccuracies in model specification.

#### 7) Standard Kaplan-Meier method

The Kaplan-Meier (KM) method offers a non-parametric approach to estimate the survival time distribution of time-to-event outcomes [44]. In this approach, we estimate the survival functions for Group 0 and Group 1, separately. This unadjusted approach was included in the numerical studies as a reference.

### Data description

#### Data generation procedure for covariates and treatment group indicator

We followed the settings from Denz et al. [36] to generate the simulation datasets. First, we generated the covariate vector $\textbf{X}=(X_1,\ldots ,X_6)^\prime .$ Specifically, $X_1 \sim \text {Bernoulli}(0.5),$ $X_2 \sim \text {Bernoulli}(0.3 + X_3 \cdot 0.1),$ $X_3 \sim \text {Bernoulli}(0.5),$ $X_4 \sim \mathcal {N}(0, 1),$ $X_5 \sim 0.3 + X_6 \cdot 0.1 + \mathcal {N}(0, 1),$ and $X_6 \sim \mathcal {N}(0, 1),$ where $\mathcal {N}(0,1)$ denotes the standard normal distribution. We then generated the treatment group indicator *Z* from a Bernoulli distribution with probability $g(a) = \exp (a)/\left\{ 1+\exp (a)\right\} ,$ where

$$\begin{aligned} a = -1.2 + \log (3) \cdot X_2 + \log (1.5) \cdot X_3 + \log (1.5) \cdot X_5 + \log (2) \cdot X_6. \end{aligned}$$

#### Data generation procedure for event time

Let *U* denote a random variable that follows a uniform distribution ranged from 0 to 1. For the event time *T*, the following two scenarios were considered:

$$\begin{aligned} & \text {Scenario 1:}\ T\\ & = \left( \frac{-\log U}{\exp (\log (1.8) \cdot X_1 + \log (1.8) \cdot X_2 + \log (1.8) \cdot X_4 + \log (2.3) \cdot X_5^2 - 1 \cdot Z)} \right) ^{0.5} \\ & \text {Scenario 2:}\ T\\ & = \left( \frac{-\log U}{\exp (\log (1.8) \cdot X_1 + \log (1.8) \cdot X_2 + \log (1.8) \cdot X_4 + \log (2.3) \cdot X_5^2 - 1 \cdot Z)} \right) . \end{aligned}$$

**Fig. 1:**
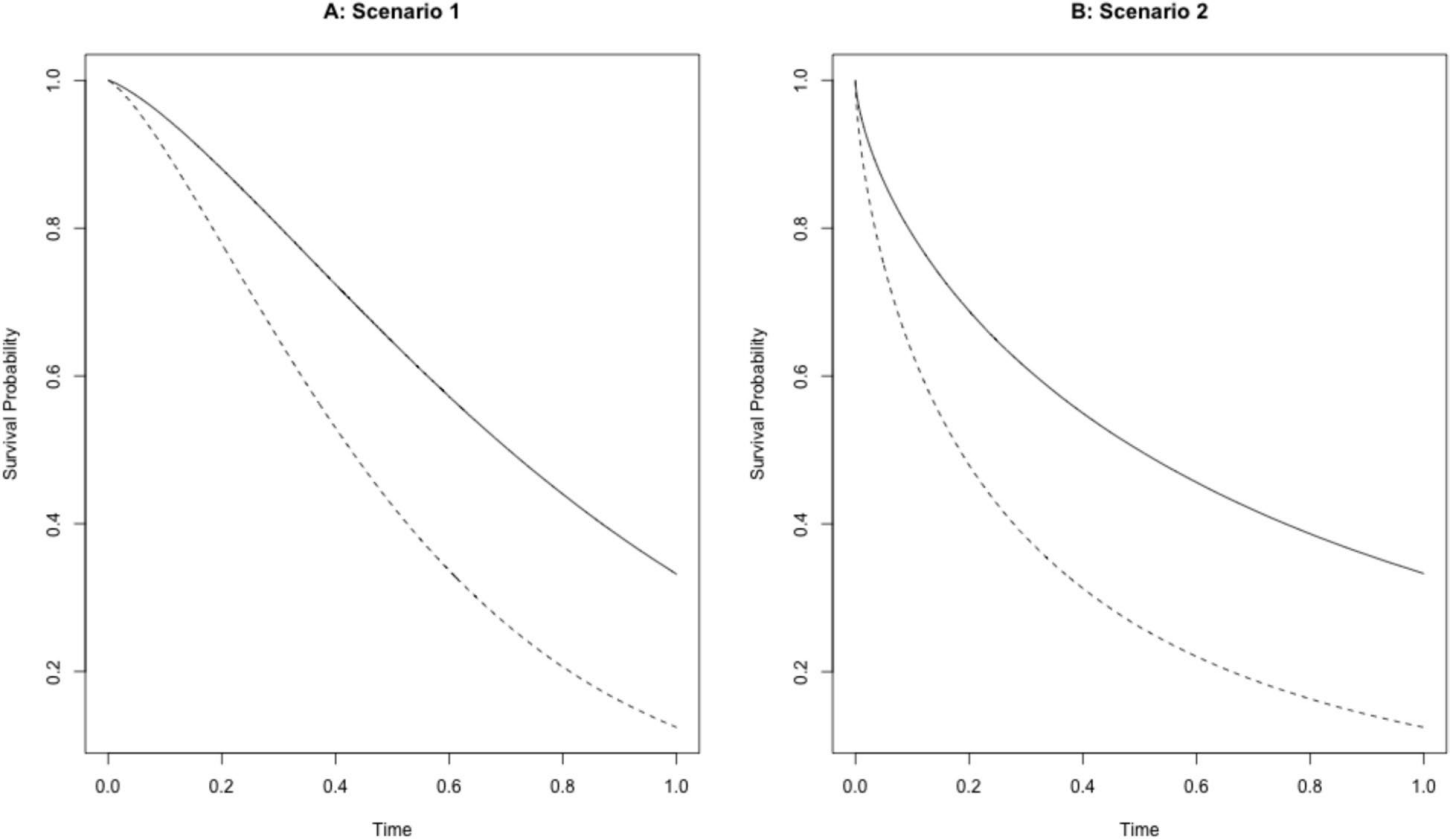
Marginal survival time distributions for Group 0 ($Z=0$, dashed line) and Group 1 ($Z=1$, solid line) in (**A**) Scenario 1 and (**B**) Scenario 2

Note that conditioned on the covariate vector $\textbf{X}$, the event times for the two groups (i.e., $Z=0$ and $Z=1$) follow Weibull distributions under Scenario 1 and exponential distributions under Scenario 2, respectively. Figure 1 presents the marginal survival time distributions for two groups under Scenarios 1 and 2.

#### Data generation procedure for censoring time

The censoring time, *C*, was generated independently of *T*. Three censoring patterns were considered as follows:

$$\begin{aligned} \text {Censoring Pattern A:}\ C & = \min (C^*, 1.0), C^* \sim \text {Exp}(0.1) \\ \text {Censoring Pattern B:}\ C & = \min (C^*, 1.0), C^* \sim \text {Exp}(0.2), \\ \text {Censoring Pattern C:}\ C & = \min (C^*, 1.0), C^* \sim \text {Exp}(0.01 + 0.4 X_2), \end{aligned}$$

where $\text {Exp}(\lambda )$ denotes the exponential distribution with the rate parameter $\lambda .$ Censoring Patterns A and B were for scenarios with completely random censoring, that is, censoring independent of both treatment and covariates. Censoring Pattern C was for covariate-dependent censoring.

#### Derivation of analysis datasets

For a given sample size *n*, we generated $\{(T_i,C_i,\textbf{X}_i,Z_i); i=1,\ldots ,n\}$ and calculated the observable data $\{(Y_i,\Delta _i,\textbf{X}_i,Z_i); i=1,\ldots ,n\},$ where $Y_i = \min (T_i,C_i),$ and $\Delta _i$ was 1 if $T_i \le C_i$ and 0 otherwise. For each simulation configuration, we generated 2000 sets of $\{(Y_i,\Delta _i,\textbf{X}_i,Z_i); i=1,\ldots ,n\}$ to evaluate the statistical performance of the methods described in Confounding adjustment methods outline section.

### Analyses

#### Cases

Most of the methods introduced in Confounding adjustment methods outline section require users to specify either the outcome model, the treatment model, or both. Although it is preferred that the correct model is specified, this is not always the case in practice. Therefore, we considered the following five different cases regarding model specification by users.

##### Case 1: Both models correct

In Case 1, we assumed that both the outcome model and treatment model were correctly specified. Let $h(t | Z, \textbf{X})$ be the hazard function for the event time *T*, given *Z* and $\textbf{X},$ and let $E[I(Z = 1) | \textbf{X}]$ denote the expected probability of getting the treatment for the subject with the covariate vector $\textbf{X}.$ The outcome model and treatment model in Case 1 are then given by

$$\begin{aligned} h(t | Z, \textbf{X}) & = h_0(t) \exp (\beta _0 Z + \beta _1 X_1 + \beta _2 X_2 + \beta _4 X_4 + \beta _5 X_5^2), \\ E[I(Z = 1) | \textbf{X}] & = g(\alpha _0 + \alpha _2 X_2 + \alpha _3 X_3 + \alpha _5 X_5 + \alpha _6 X_6), \end{aligned}$$

respectively, where $h_0(t)$ is the baseline hazard function.

##### Case 2: Both models wrong by adding extra variables

In Case 2, we assumed that users included extra variables in both outcome and treatment models, and thus neither the outcome model nor the treatment model was correctly specified. Specifically, the outcome model included $X_3$ and $X_6$, although these were not associated with the outcome. The treatment model included $X_1$ and $X_4$ that were not associated with *Z*.

$$\begin{aligned} h(t | Z, \textbf{X}) & = h_0(t) \exp (\beta _0 {Z}+\beta _1 X_1 + \beta _2 X_2 \\&+ \beta _3 X_3 + \beta _4 X_4 + \beta _5 X_5^2 + \beta _6 X_6), \\ E[I(Z = 1) | \textbf{X}] & = g(\alpha _0 + \alpha _1 X_1 + \alpha _2 X_2 + \alpha _3 X_3 \\&+ \alpha _4 X_4 + \alpha _5 X_5 + \alpha _6 X_6). \end{aligned}$$

##### Case 3: Wrong outcome model and correct treatment model

In Case 3, we assumed that users misspecified the outcome model with $X_2$ omitted but were able to specify the treatment model correctly.

$$\begin{aligned} h(t | Z, \textbf{X}) & = h_0(t) \exp (\beta _0 {Z} + \beta _1 X_1 + \beta _4 X_4 + \beta _5 X_5^2) \\ E[I(Z = 1) | \textbf{X}] & = g(\alpha _0 + \alpha _2 X_2 + \alpha _3 X_3 + \alpha _5 X_5 + \alpha _6 X_6). \end{aligned}$$

##### Case 4: Correct outcome model and wrong treatment model

In Case 4, we assumed that users correctly specified the outcome model but misspecified the treatment model with $X_2$ omitted.

$$\begin{aligned} h(t | Z, \textbf{X}) & = h_0(t) \exp (\beta _0 {Z} + \beta _1 X_1 + \beta _2 X_2 + \beta _4 X_4 + \beta _5 X_5^2) \\ E[I(Z = 1) | \textbf{X}] & = g(\alpha _0 + \alpha _3 X_3 + \alpha _5 X_5 + \alpha _6 X_6). \end{aligned}$$

##### Case 5: Both models wrong by variable omission

In Case 5, we assumed that users omitted $X_2$ from the outcome model and the treatment model.

$$\begin{aligned} h(t | Z, \textbf{X}) & = h_0(t) \exp (\beta _0 {Z} + \beta _1 X_1 + \beta _4 X_4 + \beta _5 X_5^2 )\\ E[I(Z = 1) | \textbf{X}] & = g(\alpha _0 + \alpha _3 X_3 + \alpha _5 X_5 + \alpha _6 X_6). \end{aligned}$$

Note that, regarding model properties, neither a treatment model nor an outcome model is used for KM. For DS, only outcome model is used. For IPTW KM, IPTW CH, and Matching, only treatment model is used. For EL, neither a treatment model nor an outcome model is used, but the covariates specified in treatment model will be used to estimate the likelihood in our numerical studies. For AIPTW, both outcome and treatment models are used.

#### Population parameters of interest and statistical inference

Our analyses focused on four key parameters to evaluate the AH with a truncation time $\tau =0.7$: ${\eta }_1(\tau )$, ${\eta }_0(\tau )$, ${\eta }_1(\tau )-{\eta }_0(\tau )$, and ${\eta }_1(\tau )/{\eta }_0(\tau )$, representing the AH in Group 1 and Group 0, DAH, and RAH, respectively. We estimated these parameters based on the adjusted survival curves, $\hat{S}_1(t)$ and $\hat{S}_0(t),$ derived through the methods described in Confounding adjustment methods outline section. We also calculated 95% confidence intervals using a bootstrap procedure. Specifically, for each dataset, we generated a bootstrap sample and reran the entire confounding-adjustment pipeline, including model fitting, score calculation, construction of weights or matched pairs, estimation of the adjusted survival curves, and calculation of ${\eta }_1(\tau )$, ${\eta }_0(\tau )$, DAH, and RAH. This process was repeated 300 times, allowing us to compute standard errors of the point estimators and corresponding 95% confidence intervals that account for the uncertainty introduced by weighting or matching through model fitting. Note that the standard error and confidence intervals for RAH were calculated on the log-scale.

### Performance evaluation

#### Derivation of true parameter values

The true values of the four parameters (i.e., ${\eta }_1(\tau )$, ${\eta }_0(\tau )$, DAH, and RAH) under each scenario were derived numerically. Specifically, we randomly generated 10,000 data points of the covariate vectors $\textbf{X}.$ We then generated the event time *T* when $Z=1,$ using the formula in Data description section. Using the exact same $\textbf{X},$ we generated *T* when $Z=0$ in the same manner. Combining these two datasets for the treatment $(Z=1)$ and control $(Z=0)$ groups together, we derived an estimate for each of the four parameters using the standard KM approach. To assure that the Monte Carlo error was negligible, we repeated the process 100 times and averaged the estimates for each parameter. The true values for ${\eta }_1(\tau )$, ${\eta }_0(\tau )$, DAH, and RAH were 0.934, 1.728, −0.795 and 0.541 in Scenario 1, and 1.365, 2.892, −1.527 and 0.472 in Scenario 2.

#### Performance metrics

For each of these parameters, we employed a comprehensive set of metrics to assess the performance of various confounding adjustment methods when used with the AH-based approach in CER. Firstly, we calculated the mean relative bias of point estimates, providing a scaled perspective of the bias relative to the true parameter value. Secondly, we computed the square-root of the Mean Squared Error (rMSE) for the point estimates. This metric, by incorporating both bias and variance, provides a comprehensive overview of the overall estimation error. Thirdly, we assessed the coverage probability of 95% confidence interval, which reflected the proportion of times the true parameter value falls within the calculated 95% confidence interval. Fourthly, the median length of 95% confidence interval, while being robust to the outliers, was calculated to offer insight into the efficiency of the estimating procedures.

## Results

### Independent censoring patterns

We present results from DAH (Tables 1, 2, 3 and 4), one of our four AH-based parameters, with the four performance metrics mentioned above when the total sample size is $n=300$ under Censoring Patterns A and B. Results for the remaining parameters are provided in the Supplementary Appendix B (see Tables B1 to B12).

**Table 1:** Mean relative bias for DAH

Scenario	Censoring	Method	Case				
			1	2	3	4	5
1	A	KM	−0.2887	−0.2887	−0.2887	−0.2887	−0.2887
		DS	−0.0068	−0.0065	−0.1320	−0.0068	−0.1320
		IPTW KM	−0.0071	−0.0088	−0.0071	−0.1436	−0.1436
		IPTW CH	−0.0103	−0.0119	−0.0103	−0.1453	−0.1453
		Matching	−0.0002	−0.0098	−0.0002	−0.1386	−0.1386
		EL	0.0041	0.0044	0.0041	−0.1515	−0.1515
		AIPTW	−0.0082	−0.0074	−0.0090	−0.0064	−0.1459
1	B	KM	−0.2891	−0.2891	−0.2891	−0.2891	−0.2891
		DS	−0.0069	−0.0068	−0.1321	−0.0069	−0.1321
		IPTW KM	−0.0072	−0.0089	−0.0072	−0.1434	−0.1434
		IPTW CH	−0.0106	−0.0122	−0.0106	−0.1452	−0.1452
		Matching	−0.0005	−0.0103	−0.0005	−0.1389	−0.1389
		EL	0.0029	0.0045	0.0029	−0.1508	−0.1508
		AIPTW	−0.0089	−0.0082	−0.0096	−0.0067	−0.1459
2	A	KM	−0.2612	−0.2612	−0.2612	−0.2612	−0.2612
		DS	−0.0056	−0.0054	−0.1298	−0.0056	−0.1298
		IPTW KM	0.0005	−0.0017	0.0005	−0.1364	−0.1364
		IPTW CH	−0.0067	−0.0089	−0.0067	−0.1414	−0.1414
		Matching	0.0032	−0.0019	0.0032	−0.1315	−0.1315
		EL	0.0089	0.0106	0.0089	−0.1451	−0.1451
		AIPTW	−0.0013	−0.0010	−0.0022	0.0001	−0.1397
2	B	KM	−0.2623	−0.2623	−0.2623	−0.2623	−0.2623
		DS	−0.0061	−0.0062	−0.1305	−0.0061	−0.1305
		IPTW KM	−0.0010	−0.0032	−0.0010	−0.1376	−0.1376
		IPTW CH	−0.0084	−0.0105	−0.0084	−0.1427	−0.1427
		Matching	0.0017	−0.0037	0.0017	−0.1327	−0.1327
		EL	0.0070	0.0084	0.0070	−0.1463	−0.1463
		AIPTW	−0.0024	−0.0020	−0.0032	−0.0007	−0.1405

Methods for deriving survival curves: KM, Standard Kaplan-Meier based approach (unadjusted); DS, Direct Standardization via a Cox model (G-formula); IPTW KM, Xie and Liu’s approach; IPTW CH, Cole and Hernan’s approach; Matching, Propensity score matching; EL, Empirical Likelihood approach; AIPTW, Augmented Inverse Probability of Treatment Weighting approach

**Table 2:** Square-root of Mean Squared Error (rMSE) for DAH

Scenario	Censoring	Method	Case				
			1	2	3	4	5
1	A	KM	0.2928	0.2928	0.2928	0.2928	0.2928
		DS	0.1144	0.1203	0.1562	0.1144	0.1562
		IPTW KM	0.2064	0.1999	0.2064	0.2302	0.2302
		IPTW CH	0.2034	0.1968	0.2034	0.2289	0.2289
		Matching	0.2352	0.2313	0.2352	0.2531	0.2531
		EL	0.2580	0.2355	0.2580	0.2489	0.2489
		AIPTW	0.1697	0.1704	0.1707	0.1632	0.2003
1	B	KM	0.2953	0.2953	0.2953	0.2953	0.2953
		DS	0.1156	0.1220	0.1572	0.1156	0.1572
		IPTW KM	0.2100	0.2041	0.2100	0.2331	0.2331
		IPTW CH	0.2070	0.2008	0.2070	0.2318	0.2318
		Matching	0.2397	0.2360	0.2397	0.2561	0.2561
		EL	0.2617	0.2379	0.2617	0.2504	0.2504
		AIPTW	0.1792	0.1800	0.1802	0.1728	0.2080
2	A	KM	0.5207	0.5207	0.5207	0.5207	0.5207
		DS	0.2352	0.2454	0.3064	0.2352	0.3064
		IPTW KM	0.3780	0.3631	0.3780	0.4216	0.4216
		IPTW CH	0.3713	0.3564	0.3713	0.4207	0.4207
		Matching	0.4367	0.4234	0.4367	0.4672	0.4672
		EL	0.4610	0.4345	0.4610	0.4527	0.4527
		AIPTW	0.3142	0.3155	0.3164	0.3057	0.3707
2	B	KM	0.5243	0.5243	0.5243	0.5243	0.5243
		DS	0.2373	0.2482	0.3086	0.2373	0.3086
		IPTW KM	0.3827	0.3686	0.3827	0.4260	0.4260
		IPTW CH	0.3759	0.3617	0.3759	0.4250	0.4250
		Matching	0.4404	0.4303	0.4404	0.4715	0.4715
		EL	0.4653	0.4388	0.4653	0.4561	0.4561
		AIPTW	0.3236	0.3248	0.3256	0.3143	0.3777

Methods for deriving survival curves: KM, Standard Kaplan-Meier based approach (unadjusted); DS, Direct Standardization via a Cox model (G-formula); IPTW KM, Xie and Liu’s approach; IPTW CH, Cole and Hernan’s approach; Matching, Propensity score matching; EL, Empirical Likelihood approach; AIPTW, Augmented Inverse Probability of Treatment Weighting approach

**Table 3:** Coverage probability of 95% confidence interval for DAH

Scenario	Censoring	Method	Case				
			1	2	3	4	5
1	A	KM	0.764	0.764	0.764	0.764	0.764
		DS	0.954	0.955	0.847	0.954	0.847
		IPTW KM	0.954	0.950	0.954	0.912	0.912
		IPTW CH	0.955	0.949	0.955	0.909	0.909
		Matching	0.947	0.953	0.947	0.924	0.924
		EL	0.956	0.971	0.956	0.916	0.916
		AIPTW	0.953	0.954	0.955	0.952	0.899
1	B	KM	0.769	0.769	0.769	0.769	0.769
		DS	0.955	0.954	0.858	0.955	0.858
		IPTW KM	0.957	0.948	0.957	0.915	0.915
		IPTW CH	0.956	0.948	0.956	0.915	0.915
		Matching	0.945	0.952	0.945	0.926	0.926
		EL	0.960	0.968	0.960	0.925	0.925
		AIPTW	0.951	0.949	0.954	0.951	0.903
2	A	KM	0.774	0.774	0.774	0.774	0.774
		DS	0.953	0.954	0.847	0.953	0.847
		IPTW KM	0.953	0.956	0.953	0.907	0.907
		IPTW CH	0.953	0.954	0.953	0.901	0.901
		Matching	0.951	0.955	0.951	0.924	0.924
		EL	0.965	0.969	0.965	0.914	0.914
		AIPTW	0.956	0.955	0.954	0.954	0.894
2	B	KM	0.778	0.778	0.778	0.778	0.778
		DS	0.955	0.957	0.851	0.955	0.851
		IPTW KM	0.954	0.954	0.954	0.909	0.909
		IPTW CH	0.954	0.951	0.954	0.901	0.901
		Matching	0.949	0.953	0.949	0.922	0.922
		EL	0.963	0.970	0.963	0.911	0.911
		AIPTW	0.960	0.964	0.961	0.959	0.895

Methods for deriving survival curves: KM, Standard Kaplan-Meier based approach (unadjusted); DS, Direct Standardization via a Cox model (G-formula); IPTW KM, Xie and Liu’s approach; IPTW CH, Cole and Hernan’s approach; Matching, Propensity score matching; EL, Empirical Likelihood approach; AIPTW, Augmented Inverse Probability of Treatment Weighting approach

**Table 4:** Median Length of 95% Confidence Interval for DAH

Scenario	Censoring	Method	Case				
			1	2	3	4	5
1	A	KM	0.7178	0.7178	0.7178	0.7178	0.7178
		DS	0.4484	0.4713	0.4543	0.4484	0.4543
		IPTW KM	0.7873	0.7529	0.7873	0.7685	0.7685
		IPTW CH	0.7774	0.7414	0.7774	0.7599	0.7599
		Matching	0.9087	0.9031	0.9087	0.8835	0.8835
		EL	0.9368	0.9884	0.9368	0.8293	0.8293
		AIPTW	0.6530	0.6574	0.6541	0.6375	0.6403
1	B	KM	0.7310	0.7310	0.7310	0.7310	0.7310
		DS	0.4580	0.4810	0.4645	0.4580	0.4645
		IPTW KM	0.8019	0.7679	0.8019	0.7822	0.7822
		IPTW CH	0.7910	0.7557	0.7910	0.7734	0.7734
		Matching	0.9234	0.9189	0.9234	0.8974	0.8974
		EL	0.9487	1.0008	0.9487	0.8409	0.8409
		AIPTW	0.6887	0.6937	0.6910	0.6723	0.6762
2	A	KM	1.3402	1.3402	1.3402	1.3402	1.3402
		DS	0.9288	0.9737	0.9256	0.9288	0.9256
		IPTW KM	1.4803	1.4102	1.4803	1.4351	1.4351
		IPTW CH	1.4536	1.3826	1.4536	1.4135	1.4135
		Matching	1.7005	1.6849	1.7005	1.6533	1.6533
		EL	1.7243	1.8030	1.7243	1.5272	1.5272
		AIPTW	1.2391	1.2447	1.2458	1.2139	1.2054
2	B	KM	1.3552	1.3552	1.3552	1.3552	1.3552
		DS	0.9387	0.9854	0.9361	0.9387	0.9361
		IPTW KM	1.4979	1.4274	1.4979	1.4509	1.4509
		IPTW CH	1.4688	1.3992	1.4688	1.4283	1.4283
		Matching	1.7194	1.7007	1.7194	1.6695	1.6695
		EL	1.7341	1.8135	1.7341	1.5434	1.5434
		AIPTW	1.2822	1.2881	1.2861	1.2547	1.2475

Methods for deriving survival curves: KM, Standard Kaplan-Meier based approach (unadjusted); DS, Direct Standardization via a Cox model (G-formula); IPTW KM, Xie and Liu’s approach; IPTW CH, Cole and Hernan’s approach; Matching, Propensity score matching; EL, Empirical Likelihood approach; AIPTW, Augmented Inverse Probability of Treatment Weighting approach

Table 1 presents relative bias for DAH given different confounding adjustment methods under various scenarios, censoring patterns, and cases. KM showed large bias (ranging from −0.29 to −0.26) in all situations, due to the lack of confounding adjustment. The relative bias by DS was low (around −0.006) when the outcome model was correctly specified (Case 1 and 4), but it was remarkable (around −0.13) when the outcome model failed to include a predictor (Case 3 and 5). For Case 2, where the outcome model included extra variables, the relative bias was almost identical to the ones in Case 1 and 4. This suggests that the relative bias by the DS method is more significantly affected by omitting covariates compared to adding extra covariates. Since the model used for DS is nonlinear, covariate omission generally leads to attenuation of treatment effects. While such shrinkage may be beneficial in certain statistical settings, its implications depend on the research context. In cases 3 and 5, we observed that the attenuation was substantial enough to warrant consideration, particularly when interpreting treatment effects in the presence of unmeasured confounders. The relative bias produced by IPTW KM, IPTW CH, and Matching depended on the adequacy of the treatment model, as a treatment model is used for adjustment in these approaches. When the treatment is correct (Case 1 and 3), the resulting relative bias was small (ranged from −0.01 to 0.003). For IPTW KM and IPTW CH, the relative bias in Case 2—where two extra variables were included in the treatment model—was comparable to that in Cases 1 and 3, whereas Matching showed a modest increase in the magnitude of bias under Case 2. In contrast, when a prognostic factor $X_2$ was omitted from the treatment model (Case 4 and 5), large relative biases (ranged from −0.15 to −0.13) were observed. In EL, the covariates included in the treatment model were used for adjustment. Thus, Case 1 and 3 were the situations where all variables associated with the treatment assignment were included in the analysis, and the relative biases were negligible as expected. In the case where two extra variables were included (Case 2), the absolute value of the relative bias produced by EL was less than 0.02. Where EL failed to include a variable that was associated with the treatment assignment (Case 4 and 5), large relative biases of approximately −0.15 were observed. As AIPTW has the doubly robust property, theoretically, it would produce a consistent estimate when either the outcome model or the treatment model is correctly specified (Case 1, 3, and 4). As expected, the absolute values of the relative bias in these cases were at most 0.01 in our simulation scenarios. Similar to the other methods, Case 2 produced similar results. However, when neither model was correct (Case 5), AIPTW produced large relative biases (around −0.14).

Table 2 presents the results of the rMSE for DAH. Analogous to the findings related to relative bias, the rMSE was significantly influenced by the appropriateness of the specified model(s) for each method. For instance, the DS method relies on the outcome model. The rMSE values were lower in cases where the outcome model was correctly specified (Case 1 or 4) or included all necessary predictors (Case 2), compared to cases where the outcome model omitted a crucial predictor. In Case 1, where both the outcome and treatment models were correct, the DS method provided the lowest rMSE compared to other methods. Specifically, under Scenario 1 with Censoring Pattern A and Case 1, the rMSE of DS was 0.114, while the rMSE of the other adjusted methods ranged from 0.170 to 0.258. The rMSE of Matching (0.235) was larger than the other propensity score adjustment methods (0.206 with IPTW KM and 0.203 with IPTW CH). The EL method provided the largest rMSE (0.258) among these adjusted approaches.

Table 3 presents coverage probability of 95% confidence interval for DAH given different confounding adjustment methods under various scenarios, censoring patterns, and cases. KM provided lower coverage probabilities (around 0.77) than the nominal level in all situations, due to the lack of confounding adjustment. DS had low coverage probabilities under Case 3 and 5, where the outcome model failed to include a predictor. Otherwise, the coverage probabilities of DS were closer to the nominal level. The coverage probabilities of IPTW CH, IPTW KM, and Matching were also off from the nominal level when the treatment model did not include a crucial variable (Case 4 and 5). The coverage probabilities of EL ranged from 0.956 to 0.965, which were close to the nominal level, when a correct set of variables were specified for the adjustment (Case 1 and 3). When extra variables were included for adjustment (Case 2), the coverage probabilities were higher (around 0.97) than the nominal level. When a crucial variable was not included for adjustment (Case 4 and 5), EL had low coverage probabilities (0.91 to 0.93). AIPTW performs well under Cases 1, 3, and 4 due to its doubly robust property. When either the outcome model or the treatment model is correctly specified, the coverage probabilities of AIPTW are close to the nominal level. Case 2 yields similar results. However, when neither model is correctly specified (Case 5), the coverage probability of AIPTW deviates from the nominal level (around 0.90).

Table 4 presents median length of the confidence interval for DAH. Overall, the median length of DS was shorter than those of the other methods. For example, in Case 1 under Scenario 2 with Censoring Pattern A, the median length of DS was 0.93, while the lengths of the other adjusting methods ranged from 1.24 to 1.72. The Matching and EL methods produced relatively wider confidence intervals compared to the other adjusting methods. For example, in Case 1 under Scenario 2 with Censoring Pattern B, the median lengths of Matching and EL were 1.72 and 1.73, respectively, while the lengths of the other adjusting methods ranged from 0.94 to 1.50.

Similar results were observed for the other parameters, including AH in Group 0 and Group 1 and RAH (see Supplementary Appendix Table B1 to B12).

### Covariate-dependent censoring pattern

The assumptions regarding the censoring time distribution differ across the seven methods included in our numerical study (summarized in Supplementary Appendix A). Direct standardization employs a Cox regression model as the outcome model. While this approach requires correct specification of the outcome model for asymptotic unbiasedness, it does not require the independent censoring assumption of Censoring Patterns A and B. Instead, censoring may depend on variables included in the outcome model, provided that event time and censoring time are independent conditional on those variables, as in Censoring Pattern C.

In contrast, Censoring Pattern C does not satisfy the censoring assumptions of several other methods considered. The AIPTW approach we employed in this study incorporates both an outcome model and a treatment model and is doubly robust in the sense that it achieves asymptotic unbiasedness if either the outcome model or the treatment model is correctly specified provided that the censoring mechanism is also correctly modeled. Because censoring is handled via inverse probability of censoring weights (IPCW), consistency of AIPTW further relies on a correct model for the censoring time distribution used to construct these weights, while this requirement can be a little relaxed with alternative AIPTW implementations [41, 42].

In this study, our AIPTW implementation accommodates covariate-dependent censoring by estimating the censoring distribution with a Cox model when appropriate. Specifically, in simulations with independent censoring (Censoring Patterns A and B) we used the pooled Kaplan–Meier (KM) estimator to construct IPCW; in simulations with covariate-dependent censoring (Censoring Pattern C) we used a Cox proportional hazards model for censoring that included the true covariate driving censoring. Thus, the censoring model was correctly specified in all simulation scenarios for AIPTW.

We conducted numerical studies under covariate-dependent censoring (Censoring Pattern C) to assess the impact of censoring assumptions on performance across methods. Results for total sample size $n=300$ are presented in Supplementary Appendix C (Tables C1–C16). Overall, the simulation results under Censoring Pattern C were very similar to those obtained under Censoring Patterns A and B, and our main findings were robust to this form of covariate-dependent censoring; as expected, coverage for methods relying on independent censoring (IPTW-KM/CH, Matching, EL) declines primarily when their own working model is misspecified, mirroring Patterns A and B, while AIPTW remains near nominal when its censoring model is correctly specified.

### Summary

The results of this simulation study suggest two primary insights. First, when there is confidence in the correct specification of the outcome model, DS emerges as the optimal method for confounding adjustment due to its consistently stable performance across our experiments. If the outcome model’s specification is uncertain, then AIPTW is generally recommended. AIPTW is preferred over approaches that only use either a treatment model or an outcome model due to its doubly robust property, which is particularly advantageous given the similar efficiency reflected by the median length of confidence intervals. Second, despite the theoretical expectations of EL due to its robust property, it did not perform well within the framework of our numerical studies. We found that across all assessed performance measures, AIPTW was generally superior to EL under our simulation settings.

## Example: the CANVAS trial

To illustrate the methods explored in this study, we utilized data from the pragmatic effectiveness CANVAS trial (NCT02744092; AFT-28) comparing DOACs and LMWH in preventing recurrent VTE among cancer patients with an initial VTE event. This study was conducted across 67 oncology practices in the US, and had a hybrid design with a randomization cohort and a preference cohort. A total of 671 patients consented to randomization and were randomized to receive either DOACs or LMWH. Another 140 patients declined randomization and were placed in the preference cohort where they chose either DOACs or LMWH. Of the 140, three patients did not receive the selected treatment. The primary outcome was recurrent VTE. Major bleeding and death were secondary outcomes. The detailed results with the randomization cohort data have been reported in Schrag et al. [35].

**Fig. 2:**
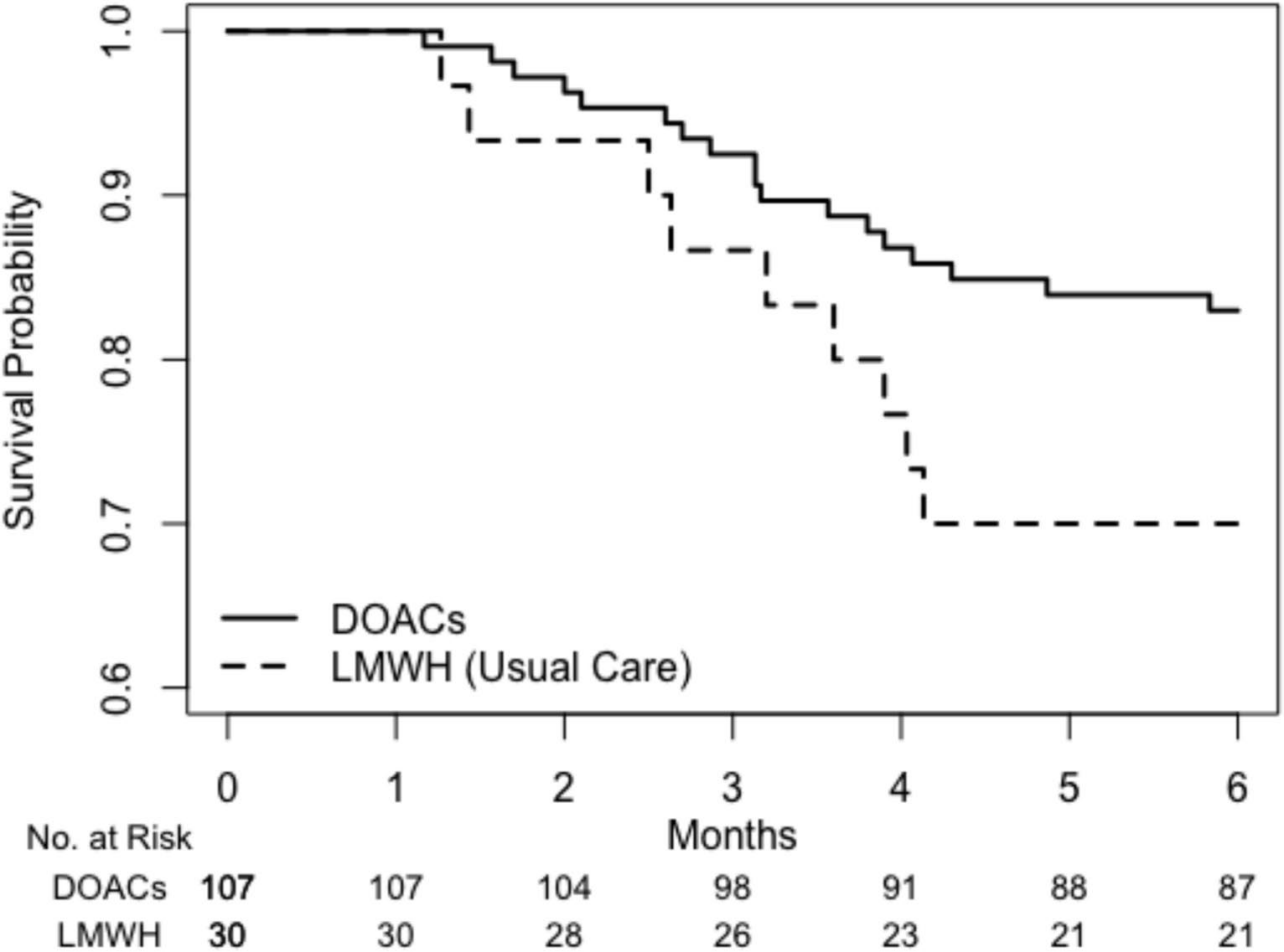
Estimated survival curves for DOAC (solid line) and LMWH (dashed line) using the Kaplan-Meier method

In this paper, we used the overall survival data from the 137 patients who received the selected treatment in the preference cohort. Figure 2 shows the unadjusted survival curves for the DOAC and LMWH groups based on the KM method. The estimated survival probabilities for the DOAC group were uniformly higher than those of the LMWH group throughout the observation period. The unadjusted AH values for the DOAC and LMWH groups, derived from the Kaplan–Meier plug-in method, were 0.031 (95% CI: 0.016–0.046) and 0.059 (95% CI: 0.019–0.099.019.099) per person-month, respectively. This indicates that the average number of events per 100 person-months would be 3.1 and 5.9 in the DOAC and LMWH groups, respectively, over the six-month study period, assuming no censored observations occur prior to six months.

In the unadjusted analysis, while the DOAC group seemed to perform better than the LMWH group, this difference was not statistically significant. Specifically, the estimated difference in AH was −0.028, with a corresponding 95% CI ranging from −0.071 to 0.015, which included zero.

Due to the lack of randomization in the treatment allocation for patients within the preference cohort, the unadjusted analysis was potentially influenced by selection bias. Thus, we performed the adjustment methods outlined in previous sections. For the execution of these adjusted analyses, we derived an outcome model and a treatment model. The details of these models are presented in Table 5 for the outcome model and Table 6 for the treatment model, respectively.

**Table 5:** Outcome model: Cox regression

Factor	Hazard Ratio	0.95 CI	*p*-value
Treatment (DOACs vs LMWH)	0.70	(0.31, 1.60)	0.397
Sex (Female vs Male)	2.95	(1.21, 7.19)	0.017
Albumin (high vs low)	0.23	(0.09, 0.56)	0.001
Metastases (Yes vs No)	2.97	(0.85, 10.38)	0.088
Pulmonary Embolism (Yes vs No)	0.58	(0.26, 1.31)	0.190

**Table 6:** Treatment model (propensity score model): logistic regression

Factor	Odds Ratio	0.95 CI	*p*-value
(Intercept)	6.10	(2.48, 14.98)	< 0.001
Sex (Female vs Male)	0.50	(0.20, 1.24)	0.134
Indwelling central venous (Yes vs No)	3.32	(1.22, 9.03)	0.019
Metastases (Yes vs No)	0.39	(0.14, 1.12)	0.080
Bevacizumab (Yes vs No)	0.27	(0.08, 0.94)	0.039

Table 7 presents both unadjusted and adjusted AH, based on pre-selected sets of adjustment variables, with a truncation time of 6 months. For example, the AH values adjusted by the AIPTW method were 0.032 per person-month for the DOAC group and 0.046 per person-month for the LMWH group. Difference in the adjusted AH was −0.014 per person-month (95% CI: −0.048 to 0.020). All methods for adjusting confounders yielded numerically similar results. These approaches uniformly indicated that, in the preference cohort, after adjustment for confounding factors, DOAC does not offer a significant survival benefit over LMWH. This outcome aligns with the observations from the randomized cohort study.

**Table 7:** Unadjusted and adjusted AH ($\tau = 6$ months) with the CANVAS mortality data from the preference cohort

Method	DOAC (0.95 CI)	LMWH (0.95 CI)	Difference (0.95 CI)	Ratio (0.95 CI)
DS	0.034 (0.018 to 0.050)	0.046 (0.019 to 0.073)	−0.012 (−0.042 to 0.018)	0.741 (0.346 to 1.586)
IPTW KM	0.030 (0.016 to 0.045)	0.049 (0.015 to 0.083)	−0.019 (−0.056 to 0.019)	0.621 (0.241 to 1.602)
IPTW CH	0.030 (0.016 to 0.045)	0.048 (0.015 to 0.081)	−0.018 (−0.054 to 0.018)	0.632 (0.247 to 1.619)
Matching	0.031 (0.016 to 0.045)	0.057 (0.013 to 0.101)	−0.027 (−0.073 to 0.020)	0.536 (0.185 to 1.555)
EL	0.030 (0.013 to 0.048)	0.056 (0.019 to 0.092)	−0.025 (−0.065 to 0.015)	0.549 (0.129 to 2.341)
AIPTW	0.032 (0.017 to 0.047)	0.046 (0.015 to 0.077)	−0.014 (−0.048 to 0.020)	0.690 (0.278 to 1.716)
KM (Unadjusted)	0.031 (0.016 to 0.046)	0.059 (0.019 to 0.099)	−0.028 (−0.071 to 0.015)	0.523 (0.208 to 1.316)

## Discussion

We confirmed that existing methods for deriving adjusted survival curves reasonably worked with the AH method in CER. If the outcome model is correct, the DS method will give consistent results. If the treatment model is correct, methods based on propensity score will give consistent results. If either outcome or treatment model is correct, AIPTW has decent performance. This conclusion is consistent with those obtained by Denz et al. [36] for other summary measures defined as a function of the survival function, such as RMST or t-year event rate.

Median survival time is also a robust summary metric of event time distribution, and difference and ratio of median could be used for summarizing the magnitude of the treatment effect. Although assessing the median survival time using confounding adjustment approaches was beyond our scope, we expect the confounding adjustment approach through the adjusted survival curves would also work well for median survival time. It would be worth conducting similar numerical studies to confirm the statistical performance of the inference procedures for the median under various settings. However, it is important to note that the median is not always estimable nonparametrically, as demonstrated in the use case example. In contrast, similar to the RMST-based approach, the AH-based approach remains applicable even when the median is inestimable.

In our simulation studies, we employed Cox regression models for the outcome model. However, other models that can provide adjusted survival curves for each group, such as accelerated failure time models or proportional odds models, can also be used. While this paper focused on methods using adjusted survival curves, if the interest lies solely in a specific metric, a regression analysis for that metric can be employed. For instance, if the estimand is an RMST-based metric, a regression analysis specific to RMST [45] can be used, although this does not provide the adjusted survival curve. Similarly, if the estimand is an AH-based metric, the regression analysis for AH recently proposed by Uno et al. [21] can also be a viable option. However, their performance relies on model assumptions, and misspecifications can lead to biased estimates. Further research is needed to systematically assess the strengths and limitations of AH regression compared to the confounding adjustment approaches examined in this paper.

When the study population can be divided into strata defined by confounding factors, stratified analyses of AH, as recently proposed by Qian et al. [22], also provide an analytic option to obtain adjusted DAH and RAH. Because $AH(\tau )$ is defined as the ratio of the cumulative incidence by $\tau$ to the RMST truncated at $\tau$ (Equation (1)), a simple weighted average of stratum-specific AHs does not generally equal the marginal $AH(\tau ).$ Likewise, the AH-based association measures DAH and RAH are generally not collapsible; that is, the marginal (crude) association does not, in general, equal a weighted average of the stratum-specific associations [46]. Qian et al. [22] developed a standardized construction of AH and studied its nonparametric inference. Specifically, let *Z* denote the treatment indicator, *X* the stratum index with $\pi _x=\Pr (X=x)$, and define $F_{jx}(\tau )={E}[I(T \le \tau )\mid Z=j, X=x]$ and $R_{jx}(\tau )={E}[\min (T,\tau )\mid Z=j, X=x]$ for $j=0,1.$ The standardized AH for group *j* is then given by $\textrm{AH}_j^{*}(\tau )=\{ \sum \nolimits _x \pi _x F_{jx}(\tau ) \} / \{ \sum \nolimits _x \pi _x R_{jx}(\tau ) \},$ which coincides with the marginal $\textrm{AH}_j(\tau ).$ The adjusted DAH and RAH are subsequently defined as $\textrm{AH}_1^{*}(\tau )-\textrm{AH}_0^{*}(\tau )$ and $\textrm{AH}_1^{*}(\tau )/\textrm{AH}_0^{*}(\tau ),$ respectively.

In this numerical study, we exclusively used bootstrapping to derive standard errors for constructing confidence intervals across all methods, even though an analytic variance formula was available for some. We did so to ensure a consistent resampling-based approach when comparing the performance of the adjustment methods. Derivation of analytic variance associated with AH for each confounding adjustment method is a promising direction for future research. Such an analytic framework could significantly reduce computational time, facilitating the execution of experiments across a broader range of scenarios.

For the matching procedure, bidirectional matching was used so that the estimand corresponds to the treatment effect in the overall study population. Standard errors were then obtained using the bootstrap procedure described in Population parameters of interest and statistical inference section, which accounts for both the sampling variability in the estimated propensity scores and the dependence induced by the matched sample. If the matched data were instead treated as independent replicates and analyzed using the standard Kaplan–Meier estimator with Greenwood’s variance formula, the resulting standard errors would be biased.

The choice of truncation time $\tau$ is central to defining the average hazard, as it specifies the time horizon over which survival is summarized. Similar to analyses based on RMST or cumulative incidence probability, $\tau$ should be selected to align with the clinical context and scientific objectives of the study, and ideally prespecified in the study protocol, especially for confirmatory analyses. When there is a prespecified follow-up duration that reflects the primary clinical question (e.g., 3-year disease-free survival or 5-year overall survival), setting $\tau$ to that time point yields an average hazard directly interpretable within that clinically meaningful window. For example, in the CANVAS trial, outcomes over a 6-month window were of primary interest, and thus $\tau =6$ months was chosen. From an analytical perspective, it is also important that sufficient numbers of patients remain at risk at $\tau$ to ensure stable estimation. For ad hoc analyses, the choice of $\tau$ should respect this constraint, whereas for prospective studies, the design should ensure that adequate follow-up is achieved at the prespecified $\tau .$

The ratio of AH is closely related to Cox’s hazard ratio, and in some special cases they coincide in estimand but differ in inference procedures. We conducted a simulation study to examine the impact of censoring rates on inference for the RAH in comparison to Cox’s HR (Supplementary Appendix D). As expected, both approaches showed negligible bias across censoring levels. When the expected size of the risk set at $\tau$ became small due to a combination of sample size and censoring rate, the AH-based approach showed somewhat reduced performance relative to Cox’s HR. This result highlights an important design consideration: ensuring an adequate risk set at the chosen truncation time is essential for stable RAH inference. Full details are provided in Supplementary Appendix D.

## Conclusions

Our study has successfully demonstrated the feasibility of applying commonly used confounding adjustment methods for survival function to AH, with all methods delivering satisfactory performance as expected. Importantly, our findings underscore the robustness of the AIPTW method, attributed to its doubly-robust property, making it a recommended approach for future research. We also identified limitations within the EL method. Despite its theoretical benefits, EL was characterized by wider confidence intervals and relatively unstable point estimates, suggesting the need for further research before its practical application can be endorsed for AH.

In summary, this study demonstrated that AH-based analysis represents a valuable addition to the survival analysis toolkit for CER. To facilitate its application, we have developed an R package that implements confounding-adjusted analyses using AH, available on GitHub (https://github.com/kkevin821/survAHadjust).

## Supplementary Information

Additional file 1: Supplementary Appendix for *“Comparative Effectiveness Research with Average Hazard for Censored Time-to-Event Outcomes: Simulation Study and Application to Observational Data”*.

## Data Availability

Program code and data supporting the findings of this study are available from the corresponding author upon reasonable request.
