# Supplementary material for: Comparative effectiveness research with average hazard for censored time-to-event outcomes: simulation study and application to observational data
Source: BMC Med Res Methodol. 2025 Dec 26;26:13. doi: 10.1186/s12874-025-02741-9 (PMC12849754; doi:10.1186/s12874-025-02741-9)
Supplement: Supplementary file 1 — Additional file 1: Supplementary Appendix for “Comparative Effectiveness Research with Average Hazard for Censored Time-to-Event Outcomes: Simulation Study and Application to Observational Data”. [file 12874_2025_2741_MOESM1_ESM.pdf]

Supplementary Appendix

for

"Comparative Effectiveness Research with Average Hazard for Censored Time-to-Event Outcomes: Simulation Study and Application to Observational Data"

Hong Xiong, Jean Connors, Deb Schrag, Hajime Uno

Contents

A Mathematical Details for Adjustment Methods **3**

B Simulation Results for AH in each group and RAH **7**

C Simulation Results with Censoring Pattern C **19**

C.1 DAH 19

C.2 AH in each group and RAH 23

D Impact of censoring rate on inference based on the Average Hazard approach and Cox's approach **35**

D.1 Simulation Configurations 35

D.2 Simulation Results 35

List of Tables

B1 Mean Relative Bias for AH in Group 0 7

B2 Square-root of Mean Squared Error (rMSE) for AH in Group 0 8

B3 Coverage Probability of 0.95 Confidence Interval for AH in Group 0 9

B4 Median Length of 0.95 Confidence Interval for AH in Group 0 10

B5 Mean Relative Bias for AH in Group 1 11

B6 Square-root of Mean Squared Error (rMSE) for AH in Group 1 12

B7 Coverage Probability of 0.95 Confidence Interval for AH in Group 1 13

B8 Median Length of 0.95 Confidence Interval for AH in Group 1 14

B9 Mean Relative Bias for log(RAH) 15

B10 Square-root of Mean Squared Error (rMSE) for log(RAH) 16

B11 Coverage Probability of 0.95 Confidence Interval for RAH 17

B12 Median Length of 0.95 Confidence Interval for log(RAH) 18

C1 Mean Relative Bias for DAH 19

C2 Square-root of Mean Squared Error (rMSE) for DAH 20

C3 Coverage Probability of 0.95 Confidence Interval for DAH 21

C4 Median Length of 0.95 Confidence Interval for DAH 22

C5 Mean Relative Bias for AH in Group 0 23
C6 Square-root of Mean Squared Error (rMSE) for AH in Group 0 24
C7 Coverage Probability of 0.95 Confidence Interval for AH in Group 0 25
C8 Median Length of 0.95 Confidence Interval for AH in Group 0 26
C9 Mean Relative Bias for AH in Group 1 27
C10 Square-root of Mean Squared Error (rMSE) for AH in Group 1 28
C11 Coverage Probability of 0.95 Confidence Interval for AH in Group 1 29
C12 Median Length of 0.95 Confidence Interval for AH in Group 1 30
C13 Mean Relative Bias for log(RAH) 31
C14 Square-root of Mean Squared Error (rMSE) for log(RAH) 32
C15 Coverage Probability of 0.95 Confidence Interval for RAH 33
C16 Median Length of 0.95 Confidence Interval for log(RAH) 34
D1 Bias, coverage probability of 95% CI, and average standard error (Ave SE) comparing Cox's HR and RAH under varying censoring rates and sample size based on 5000 iterations. 36

A Mathematical Details for Adjustment Methods

Common notation and assumptions

Let T denote the event time and C the censoring time. We observe (Y, Δ) with $Y = \min(T, C)$ and $\Delta = I(T \leq C)$. For $i = 1, \ldots, n$, let $(Y_i, \Delta_i, Z_i, X_i)$ be the observed data, where $Z_i \in \{0, 1\}$ indicates treatment ($Z_i = 1$) or control ($Z_i = 0$) and $X_i \in \mathbb{R}^p$ are baseline covariates. The n observations are assumed independent and identically distributed.

For $t \geq 0$, define the at-risk and counting processes for subject i by $Y_i(t) = I(Y_i \geq t)$ and $N_i(t) = I(Y_i \leq t,\ \Delta_i = 1)$, and let $\mathcal{T} = \{t_j : \sum_{i=1}^{n} I(Y_i = t_j,\ \Delta_i = 1) > 0\}$ be the set of distinct observed event times.

For causal inference, we invoke the potential outcomes framework. Let T^z denote the potential event time that would be observed for a subject if treatment were set to level $z \in \{0, 1\}$. The potential-outcome survival function is then $S_z(t) = P(T^z > t)$ for $t \geq 0$.

We use the propensity score $e(X) = P(Z = 1 \mid X)$ and write $e_1(X) = e(X)$ and $e_0(X) = 1 - e(X)$, with estimates $\widehat{e}_z(X)$ obtained from a parametric model [1]. In our numerical studies, we use logistic regression models.

Regarding the censoring time C, each method has slightly different assumptions. The following assumptions apply:

- **Direct standardization (DS).** DS relies on a Cox regression model for the outcome and is consistent if that model is correctly specified. Because censoring is allowed to depend on the variables included in the outcome model, the relevant assumption is $T \perp C \mid (Z, X)$.

- **Methods without an outcome model** (i.e., IPTW-KM, IPTW-CH, matching, EL weighting, and unadjusted KM). Because no censoring model is fit, these methods assume the independent censoring within the weighted or matched sample for each group, i.e., $C \perp (T, X) \mid Z$.

- **AIPTW (as implemented).** The AIPTW implementation in this study follows Equation (7) of Ozenne et al. [2] (see details below). This estimator uses inverse probability of censoring weights (IPCW) and therefore requires conditionally independent censoring and a consistent estimator of the censoring survival function: $C \perp T \mid (X, Z)$ and consistency of $\widehat{G}(t \mid X, Z) = \Pr(C > t \mid X, Z)$. Under these conditions, it is doubly robust in the sense that consistency holds if either the outcome model or the treatment model is correctly specified (given a correct G). Alternative AIPTW implementations (e.g., Equation (9) in Ozenne et al. [2] and Zhang and Schaubel [3]) can further relax the need for a correct G.

- **AIPTW (as implemented).** The AIPTW implementation in this study follows Equation (7) of Ozenne et al. [2]. This estimator uses inverse probability of censoring weights (IPCW) and requires conditionally independent censoring and a consistent estimator of the censoring survival function; that is, $C \perp T \mid (X, Z)$ and consistency of $\widehat{G}(t \mid X, Z) = \Pr(C > t \mid X, Z)$. Under these conditions, it is doubly robust in the sense that consistency holds if either the outcome model or the treatment model is correctly specified (provided G is correctly specified). Alternative AIPTW formulations that include an explicit censoring-augmentation term (e.g., Equation (9) in Ozenne et al. [2], Zhang and Schaubel [3]) can further relax the need for a correct G.

  In our simulation studies, under Censoring Patterns 1 and 2 where $C \perp T$ hold, the *pooled* Kaplan-Meier estimator was used for the censoring survival function $G(t) = \Pr(C \geq t)$ in computation of IPCW. Under Censoring pattern 3, where $C \perp T \mid (X, Z)$, we instead estimated $G(t \mid X, Z)$ using a Cox regression model.

All methods below produce adjusted survival curves $\widehat{S}_z(t)$ ($z \in \{0,1\}$). In our AH-based analyses, these are subsequently transformed to the (group-specific) average hazard with the truncation time τ via

$$\widehat{\mathrm{AH}}_z(\tau) = \frac{1-\widehat{S}_z(\tau)}{\int_0^\tau \widehat{S}_z(u)\,du}.$$

The difference and ratio of the AH are then derived by $\widehat{\mathrm{DAH}}(\tau) = \widehat{\mathrm{AH}}_1(\tau) - \widehat{\mathrm{AH}}_0(\tau)$ and $\widehat{\mathrm{RAH}}(\tau) = \widehat{\mathrm{AH}}_1(\tau)/\widehat{\mathrm{AH}}_0(\tau)$, respectively.

1) Direct Standardization (DS)

We fit a Cox proportional hazards model with covariates (Z, X):

$$\lambda(t \mid Z, X) = \lambda_0(t)\exp\{\beta Z + \gamma^\top X\}, \tag{1}$$

where $\lambda_0(t)$ is the baseline hazard function and $(\beta, \gamma^\top)^\top$ is the vector of regression coefficients. The parameters $(\widehat{\beta}, \widehat{\gamma}^\top)^\top$ are obtained by maximizing the partial likelihood, and the baseline cumulative hazard, $\Lambda_0(t) = \int_0^t \lambda_0(u)\,du$ is estimated by the Breslow estimator, $\widehat{\Lambda}_0(t)$.

For each subject i, the counterfactual survival under treatment z is predicted as

$$\widehat{S}_i^{(z)}(t) = \exp\Big\{-\widehat{\Lambda}_0(t)\exp\big(\widehat{\beta}z + \widehat{\gamma}^\top X_i\big)\Big\}.$$

The adjusted survival curve for z is given by averaging these over the empirical distribution of X:

$$\widehat{S}_z^{\mathrm{DS}}(t) = \frac{1}{n}\sum_{i=1}^n \widehat{S}_i^{(z)}(t), \qquad z \in \{0,1\}.$$

Note that interactions and nonlinear terms may also be included in (1) as specified. [4, 5, 6]

2) IPTW Kaplan–Meier (IPTW-KM)

In this paper, the approach proposed by Xie and Liu [7] was used. Specifically, the weights defined as

$$\tilde{w}_i^{\mathrm{IPTW}}(z) = \frac{I(Z_i = z)}{\widehat{e}_z(X_i)}, \tag{2}$$

were used, where $\widehat{e}_1(X_i) = \widehat{e}(X_i)$ and $\widehat{e}_0(X_i) = 1 - \widehat{e}(X_i)$.

For generic nonnegative weights a_i attached to subjects, the weighted KM estimator within the group z is

$$\widehat{S}_z^{(a)}(t) = \prod_{t_j \in \mathcal{T},\, t_j \le t}\left\{1 - \frac{\sum_{i=1}^n a_i\, I(Z_i = z)\, I(Y_i = t_j, \Delta_i = 1)}{\sum_{i=1}^n a_i\, I(Z_i = z)\, I(Y_i \ge t_j)}\right\}.$$

Note that when $a_i \equiv 1$, this reduces to the usual (per-group) Kaplan–Meier [8]. The adjusted survival curve derived from the IPTW-KM method [7] is denoted by

$$\widehat{S}_z^{\mathrm{IPTW\text{-}KM}}(t) = \widehat{S}_z^{(a)}(t) \;\text{ with }\; a_i = \tilde{w}_i^{\mathrm{IPTW}}(z).$$

3) IPTW Survival via Cumulative Hazard (IPTW-CH)

For generic nonnegative weights a_i attached to subjects, the weighted Nelson–Aalen for the cumulative hazard function within group z is

$$\widehat{\Lambda}_z^{(a)}(t) = \sum_{t_j \in \mathcal{T},\, t_j \le t} \frac{\sum_{i=1}^n a_i\, I(Z_i = z)\, I(Y_i = t_j, \Delta_i = 1)}{\sum_{i=1}^n a_i\, I(Z_i = z)\, I(Y_i \ge t_j)}.$$

Using the weights derived through the treatment model, we estimate the weighted cumulative hazard function by

$$\widehat{\Lambda}_z^{\mathrm{IPTW}}(t) = \widehat{\Lambda}_z^{(a)}(t) \;\;\text{with}\;\; a_i = \tilde{w}_i^{\mathrm{IPTW}}(z),$$

and thus,

$$\widehat{S}_z^{\text{IPTW-CH}}(t) = \exp\{-\widehat{\Lambda}_z^{\mathrm{IPTW}}(t)\}.$$

Note that this is equivalent to fitting a Cox model stratified by Z with observation weights w_i^{IPTW} and extracting group-specific baseline cumulative hazards [9].

4) Propensity Score Matching

Based the fitted treatment model, $\widehat{e}(X_i)$ is calculated for each subject in the dataset. Two sets of nearest-neighbor matches, with replacement, are then constructed:

(i) $\mathcal{M}_{1\to 0} = \{(i,j) : Z_i = 1,\ Z_j = 0,\ j = \arg\min_{k:Z_k=0}\ |\widehat{e}(X_i) - \widehat{e}(X_k)|\}$.

(ii) $\mathcal{M}_{0\to 1} = \{(i,j) : Z_i = 0,\ Z_j = 1,\ j = \arg\min_{k:Z_k=1}\ |\widehat{e}(X_i) - \widehat{e}(X_k)|\}$.

Let the matched multiplicity weight for subject i be

$$\omega_i \;=\; \#\{(i,\cdot) \in \mathcal{M}_{1\to 0} \cup \mathcal{M}_{0\to 1}\} \;+\; \#\{(\cdot,i) \in \mathcal{M}_{1\to 0} \cup \mathcal{M}_{0\to 1}\}.$$

Restrict to the matched sample $\mathcal{I}_m = \{i : \omega_i > 0\}$ and normalize within each group z by $\tilde{\omega}_i(z) = \omega_i\, I(Z_i = z) \Big/ \big(\sum_{j\in\mathcal{I}_m} \omega_j I(Z_j = z)\big)$. Estimate matched survival curves by weighted KM on $\mathcal{I}_m$:

$$\widehat{S}_z^{\mathrm{match}}(t) = \prod_{t_j \in \mathcal{T},\, t_j \le t} \left\{1 - \frac{\sum_{i\in\mathcal{I}_m} \tilde{\omega}_i(z)\, I(Y_i = t_j, \Delta_i = 1)}{\sum_{i\in\mathcal{I}_m} \tilde{\omega}_i(z)\, I(Y_i \ge t_j)}\right\}.$$

This bidirectional construction preserves representation from both arms and targets the ATE under standard matching assumptions; independent censoring is assumed in the matched sample [10].

5) Empirical Likelihood (EL) weighting

The method proposed by Wang et al. [11] was used for the EL weighting method. Let $\phi(X) \in \mathbb{R}^m$ be a user-chosen vector of balance functions (e.g., first moments $\phi(X) = X$, or first and selected second moments $\phi(X) = (X, \mathrm{vec}(XX^\top))$). Let $D_i = I(Z_i = 1)$ and define unknown empirical probabilities $\{p_i\}_{i=1}^n$.

The EL weights are obtained by maximizing the empirical likelihood subject to arm-specific normalization and moment-balance constraints:

$$\max_{p_1,\dots,p_n} \sum_{i=1}^n \log p_i \quad \text{s.t.} \quad p_i \ge 0,\ \sum_{i=1}^n p_i D_i = 1,\ \sum_{i=1}^n p_i(1-D_i) = 1,\ \sum_{i=1}^n p_i D_i\, \phi(X_i) = \sum_{i=1}^n p_i(1-D_i)\, \phi(X_i).$$

Let $n_1 = \sum_i D_i$, $n_0 = \sum_i (1 - D_i)$, and $a = E\{\phi(X)\}$ (estimated by the sample mean $\bar{\phi} = \frac{1}{n}\sum_i \phi(X_i)$). By the Lagrange multiplier solution, the optimal probabilities satisfy

$$p_i D_i \ = \ \frac{D_i}{n_1 + \gamma^\top\{\phi(X_i) - a\}}, \qquad p_i(1 - D_i) \ = \ \frac{1 - D_i}{n_0 - \gamma^\top\{\phi(X_i) - a\}},$$

where the multiplier $\gamma \in \mathbb{R}^m$ and a solve the estimating equations

$$\sum_{i=1}^{n} \frac{D_i\,\{\phi(X_i) - a\}}{n_1 + \gamma^\top\{\phi(X_i) - a\}} = 0, \qquad \sum_{i=1}^{n} \frac{(1 - D_i)\,\{\phi(X_i) - a\}}{n_0 - \gamma^\top\{\phi(X_i) - a\}} = 0.$$

With the EL weights, $\{p_i\}_{i=1}^n$, the EL-adjusted survival curve for group z is given by

$$\widehat{S}_z^{\mathrm{EL}}(t) = \prod_{t_j \in \mathcal{T},\, t_j \le t} \left\{1 - \frac{\sum_{i=1}^n p_i I(Z_i = z)\, I(Y_i = t_j, \Delta_i = 1)}{\sum_{i=1}^n p_i I(Z_i = z)\, I(Y_i \ge t_j)}\right\}.$$

6) Augmented Inverse Probability of Treatment Weighting (AIPTW)

We implemented the AIPTW approach following Ozenne et al. [2]. Let $F_z(t) = 1 - S_z(t)$ denote the cumulative incidence probability in group z at time t. Let $\widehat{\Lambda}_{\mathrm{Cox}}(t \mid z, X)$ denote the estimated cumulative hazard function from a Cox regression outcome model, and define

$$\widehat{F}_{\mathrm{Cox}}(t \mid z, X) = 1 - \exp\{-\widehat{\Lambda}_{\mathrm{Cox}}(t \mid z, X)\},$$

the corresponding model-based cumulative incidence probability.

Denote by $a_i = \tilde{w}_i^{\mathrm{IPTW}}(z)$ the IPTW, as defined in Equation (2). In addition, the AIPTW approach requires modeling of the censoring time distribution. Assume that the censoring time C may depend on both baseline covariates X and treatment Z. We then fit a model for the censoring distribution, for example, a Cox regression model. Let $\hat{\Lambda}_{\mathrm{cens}}(t|Z, X)$ denote the estimated cumulative hazard function from this censoring model. The corresponding survival function of the censoring time, $G(t \mid Z, X) = \Pr(C \ge t \mid Z, X)$, is given by

$$\widehat{G}(t \mid Z, X) = \exp\{-\widehat{\Lambda}_{\mathrm{cens}}(t \mid Z, X)\}.$$

For $z \in \{0, 1\}$, the AIPTW estimator of the cumulative incidence probability is

$$\widehat{F}_z^{\mathrm{AIPTW}}(t) = \frac{1}{n}\sum_{i=1}^{n}\left[\frac{I(Y_i \le t,\ \Delta_i = 1)}{\widehat{G}(Y_i \mid Z_i, X_i)}\, a_i \ + \ \widehat{F}_{\mathrm{Cox}}(t \mid z, X_i)\,\{1 - a_i\}\right]. \tag{3}$$

The corresponding adjusted survival function is

$$\widehat{S}_z^{\mathrm{AIPTW}}(t) = 1 - \widehat{F}_z^{\mathrm{AIPTW}}(t).$$

When the assumption of independent censoring is plausible, one may instead use the pooled Kaplan-Meier estimator $\widehat{G}(t)$ for $G(t) = \Pr(C \ge t)$, instead of $\widehat{G}(t \mid Z, X)$.

7) Unadjusted Kaplan–Meier (reference)

The unadjusted survival curve is given by

$$\widehat{S}_z^{\mathrm{KM}}(t) = \prod_{t_j \in \mathcal{T},\, t_j \le t} \left\{1 - \frac{\sum_{i=1}^n I(Z_i = z)\, I(Y_i = t_j, \Delta_i = 1)}{\sum_{i=1}^n I(Z_i = z)\, I(Y_i \ge t_j)}\right\},$$

under independent censoring within group z [8].

B Simulation Results for AH in each group and RAH

Table B1: Mean Relative Bias for AH in Group 0

			Case				
Scenario	Censoring	Method	1	2	3	4	5
1	A	KM	-0.0630	-0.0630	-0.0630	-0.0630	-0.0630
		DS	-0.0039	-0.0039	-0.0328	-0.0039	-0.0328
		IPTW KM	-0.0008	-0.0016	-0.0008	-0.0330	-0.0330
		IPTW CH	-0.0068	-0.0077	-0.0068	-0.0382	-0.0382
		Matching	-0.0008	-0.0028	-0.0008	-0.0308	-0.0308
		EL	-0.0013	-0.0010	-0.0013	-0.0349	-0.0349
		AIPTW	-0.0012	-0.0014	-0.0016	-0.0013	-0.0339
1	B	KM	-0.0631	-0.0631	-0.0631	-0.0631	-0.0631
		DS	-0.0039	-0.0039	-0.0329	-0.0039	-0.0329
		IPTW KM	-0.0011	-0.0020	-0.0011	-0.0332	-0.0332
		IPTW CH	-0.0074	-0.0084	-0.0074	-0.0387	-0.0387
		Matching	-0.0013	-0.0033	-0.0013	-0.0310	-0.0310
		EL	-0.0017	-0.0014	-0.0017	-0.0351	-0.0351
		AIPTW	-0.0020	-0.0023	-0.0023	-0.0018	-0.0343
2	A	KM	-0.0704	-0.0704	-0.0704	-0.0704	-0.0704
		DS	-0.0026	-0.0025	-0.0397	-0.0026	-0.0397
		IPTW KM	0.0040	0.0028	0.0040	-0.0372	-0.0372
		IPTW CH	-0.0048	-0.0061	-0.0048	-0.0448	-0.0448
		Matching	0.0047	0.0029	0.0047	-0.0335	-0.0335
		EL	0.0042	0.0048	0.0042	-0.0394	-0.0394
		AIPTW	0.0026	0.0024	0.0021	0.0025	-0.0390
2	B	KM	-0.0708	-0.0708	-0.0708	-0.0708	-0.0708
		DS	-0.0029	-0.0028	-0.0401	-0.0029	-0.0401
		IPTW KM	0.0035	0.0023	0.0035	-0.0377	-0.0377
		IPTW CH	-0.0056	-0.0068	-0.0056	-0.0455	-0.0455
		Matching	0.0043	0.0026	0.0043	-0.0340	-0.0340
		EL	0.0037	0.0043	0.0037	-0.0399	-0.0399
		AIPTW	0.0022	0.0021	0.0018	0.0021	-0.0393

Methods for deriving survival curves: KM, Standard Kaplan-Meier based approach (unadjusted); DS, Direct Standardization via a Cox model (G-formula); IPTW KM, Xie and Liu's approach; IPTW CH, Cole and Hernan's approach; Matching, Propensity score matching; EL, Empirical Likelihood approach; AIPTW, Augmented Inverse Probability of Treatment Weighting approach.

Case	Outcome model	Treatment model
1	Correct	Correct
2	Included extra variables, X_3 and X_6	Included extra variables, X_1 and X_4
3	Failed to include X_2	Correct
4	Correct	Failed to include X_2
5	Failed to include X_2	Failed to include X_2

Table B2: Square-root of Mean Squared Error (rMSE) for AH in Group 0

					Case		
Scenario	Censoring	Method	1	2	3	4	5
1	A	KM	0.1672	0.1672	0.1672	0.1672	0.1672
		DS	0.1143	0.1159	0.1254	0.1143	0.1254
		IPTW KM	0.1455	0.1424	0.1455	0.1506	0.1506
		IPTW CH	0.1444	0.1414	0.1444	0.1532	0.1532
		Matching	0.1656	0.1612	0.1656	0.1653	0.1653
		EL	0.1780	0.1672	0.1780	0.1553	0.1553
		AIPTW	0.1318	0.1317	0.1329	0.1302	0.1403
1	B	KM	0.1689	0.1689	0.1689	0.1689	0.1689
		DS	0.1157	0.1174	0.1268	0.1157	0.1268
		IPTW KM	0.1474	0.1447	0.1474	0.1526	0.1526
		IPTW CH	0.1463	0.1438	0.1463	0.1553	0.1553
		Matching	0.1684	0.1642	0.1684	0.1673	0.1673
		EL	0.1799	0.1692	0.1799	0.1567	0.1567
		AIPTW	0.1372	0.1372	0.1384	0.1352	0.1451
2	A	KM	0.3312	0.3312	0.3312	0.3312	0.3312
		DS	0.2416	0.2452	0.2595	0.2416	0.2595
		IPTW KM	0.3030	0.2964	0.3030	0.3066	0.3066
		IPTW CH	0.2984	0.2922	0.2984	0.3114	0.3114
		Matching	0.3446	0.3350	0.3446	0.3379	0.3379
		EL	0.3568	0.3511	0.3568	0.3153	0.3153
		AIPTW	0.2752	0.2755	0.2771	0.2732	0.2852
2	B	KM	0.3345	0.3345	0.3345	0.3345	0.3345
		DS	0.2447	0.2485	0.2626	0.2447	0.2626
		IPTW KM	0.3069	0.3005	0.3069	0.3105	0.3105
		IPTW CH	0.3023	0.2962	0.3023	0.3154	0.3154
		Matching	0.3471	0.3405	0.3471	0.3421	0.3421
		EL	0.3601	0.3549	0.3601	0.3184	0.3184
		AIPTW	0.2828	0.2831	0.2848	0.2799	0.2914

Methods for deriving survival curves: KM, Standard Kaplan-Meier based approach (unadjusted); DS, Direct Standardization via a Cox model (G-formula); IPTW KM, Xie and Liu's approach; IPTW CH, Cole and Hernan's approach; Matching, Propensity score matching; EL, Empirical Likelihood approach; AIPTW, Augmented Inverse Probability of Treatment Weighting approach.

Case	Outcome model	Treatment model
1	Correct	Correct
2	Included extra variables, X_3 and X_6	Included extra variables, X_1 and X_4
3	Failed to include X_2	Correct
4	Correct	Failed to include X_2
5	Failed to include X_2	Failed to include X_2

Table B3: Coverage Probability of 0.95 Confidence Interval for AH in Group 0

			Case				
Scenario	Censoring	Method	1	2	3	4	5
1	A	KM	0.854	0.854	0.854	0.854	0.854
		DS	0.953	0.954	0.921	0.953	0.921
		IPTW KM	0.955	0.954	0.955	0.926	0.926
		IPTW CH	0.952	0.953	0.952	0.913	0.913
		Matching	0.953	0.958	0.953	0.930	0.930
		EL	0.964	0.980	0.964	0.937	0.937
		AIPTW	0.954	0.955	0.954	0.952	0.922
1	B	KM	0.861	0.861	0.861	0.861	0.861
		DS	0.952	0.953	0.918	0.952	0.918
		IPTW KM	0.956	0.955	0.956	0.928	0.928
		IPTW CH	0.950	0.952	0.950	0.917	0.917
		Matching	0.949	0.953	0.949	0.935	0.935
		EL	0.964	0.977	0.964	0.937	0.937
		AIPTW	0.955	0.954	0.956	0.954	0.924
2	A	KM	0.848	0.848	0.848	0.848	0.848
		DS	0.951	0.955	0.907	0.951	0.907
		IPTW KM	0.956	0.955	0.956	0.919	0.919
		IPTW CH	0.950	0.949	0.950	0.907	0.907
		Matching	0.955	0.955	0.955	0.930	0.930
		EL	0.962	0.977	0.962	0.926	0.926
		AIPTW	0.951	0.952	0.951	0.952	0.917
2	B	KM	0.847	0.847	0.847	0.847	0.847
		DS	0.953	0.952	0.906	0.953	0.906
		IPTW KM	0.953	0.951	0.953	0.912	0.912
		IPTW CH	0.947	0.946	0.947	0.904	0.904
		Matching	0.954	0.958	0.954	0.929	0.929
		EL	0.960	0.975	0.960	0.924	0.924
		AIPTW	0.953	0.954	0.955	0.954	0.918

Methods for deriving survival curves: KM, Standard Kaplan-Meier based approach (unadjusted); DS, Direct Standardization via a Cox model (G-formula); IPTW KM, Xie and Liu's approach; IPTW CH, Cole and Hernan's approach; Matching, Propensity score matching; EL, Empirical Likelihood approach; AIPTW, Augmented Inverse Probability of Treatment Weighting approach.

Case	Outcome model	Treatment model
1	Correct	Correct
2	Included extra variables, X_3 and X_6	Included extra variables, X_1 and X_4
3	Failed to include X_2	Correct
4	Correct	Failed to include X_2
5	Failed to include X_2	Failed to include X_2

Table B4: Median Length of 0.95 Confidence Interval for AH in Group 0

			Case				
Scenario	Censoring	Method	1	2	3	4	5
1	A	KM	0.5147	0.5147	0.5147	0.5147	0.5147
		DS	0.4606	0.4672	0.4532	0.4606	0.4532
		IPTW KM	0.5739	0.5626	0.5739	0.5520	0.5520
		IPTW CH	0.5683	0.5566	0.5683	0.5471	0.5471
		Matching	0.6488	0.6476	0.6488	0.6293	0.6293
		EL	0.6811	0.7782	0.6811	0.5829	0.5829
		AIPTW	0.5267	0.5286	0.5293	0.5224	0.5115
1	B	KM	0.5222	0.5222	0.5222	0.5222	0.5222
		DS	0.4668	0.4731	0.4598	0.4668	0.4598
		IPTW KM	0.5832	0.5720	0.5832	0.5622	0.5622
		IPTW CH	0.5769	0.5653	0.5769	0.5569	0.5569
		Matching	0.6601	0.6597	0.6601	0.6394	0.6394
		EL	0.6895	0.7850	0.6895	0.5933	0.5933
		AIPTW	0.5485	0.5508	0.5511	0.5425	0.5305
2	A	KM	1.0554	1.0554	1.0554	1.0554	1.0554
		DS	0.9712	0.9868	0.9446	0.9712	0.9446
		IPTW KM	1.2054	1.1797	1.2054	1.1448	1.1448
		IPTW CH	1.1863	1.1599	1.1863	1.1293	1.1293
		Matching	1.3622	1.3556	1.3622	1.3051	1.3051
		EL	1.3792	1.5200	1.3792	1.1971	1.1971
		AIPTW	1.1025	1.1064	1.1084	1.0981	1.0530
2	B	KM	1.0655	1.0655	1.0655	1.0655	1.0655
		DS	0.9807	0.9969	0.9537	0.9807	0.9537
		IPTW KM	1.2196	1.1900	1.2196	1.1563	1.1563
		IPTW CH	1.2005	1.1706	1.2005	1.1399	1.1399
		Matching	1.3752	1.3678	1.3752	1.3170	1.3170
		EL	1.3893	1.5391	1.3893	1.2059	1.2059
		AIPTW	1.1308	1.1353	1.1354	1.1223	1.0768

Methods for deriving survival curves: KM, Standard Kaplan-Meier based approach (unadjusted); DS, Direct Standardization via a Cox model (G-formula); IPTW KM, Xie and Liu's approach; IPTW CH, Cole and Hernan's approach; Matching, Propensity score matching; EL, Empirical Likelihood approach; AIPTW, Augmented Inverse Probability of Treatment Weighting approach.

Case	Outcome model	Treatment model
1	Correct	Correct
2	Included extra variables, X_3 and X_6	Included extra variables, X_1 and X_4
3	Failed to include X_2	Correct
4	Correct	Failed to include X_2
5	Failed to include X_2	Failed to include X_2

Table B5: Mean Relative Bias for AH in Group 1

Scenario	Censoring	Method	Case 1	2	3	4	5
1	A	KM	0.1292	0.1292	0.1292	0.1292	0.1292
		DS	-0.0015	-0.0017	0.0517	-0.0015	0.0517
		IPTW KM	0.0046	0.0045	0.0046	0.0612	0.0612
		IPTW CH	-0.0038	-0.0041	-0.0038	0.0530	0.0530
		Matching	-0.0013	0.0032	-0.0013	0.0610	0.0610
		EL	-0.0059	-0.0057	-0.0059	0.0643	0.0643
		AIPTW	0.0047	0.0037	0.0047	0.0030	0.0615
1	B	KM	0.1293	0.1293	0.1293	0.1293	0.1293
		DS	-0.0013	-0.0015	0.0515	-0.0013	0.0515
		IPTW KM	0.0041	0.0038	0.0041	0.0605	0.0605
		IPTW CH	-0.0047	-0.0051	-0.0047	0.0520	0.0520
		Matching	-0.0019	0.0027	-0.0019	0.0608	0.0608
		EL	-0.0056	-0.0065	-0.0056	0.0634	0.0634
		AIPTW	0.0039	0.0028	0.0039	0.0024	0.0608
2	A	KM	0.1431	0.1431	0.1431	0.1431	0.1431
		DS	0.0007	0.0007	0.0610	0.0007	0.0610
		IPTW KM	0.0078	0.0079	0.0078	0.0737	0.0737
		IPTW CH	-0.0028	-0.0029	-0.0028	0.0633	0.0633
		Matching	0.0064	0.0082	0.0064	0.0761	0.0761
		EL	-0.0011	-0.0016	-0.0011	0.0788	0.0788
		AIPTW	0.0069	0.0063	0.0069	0.0051	0.0737
2	B	KM	0.1434	0.1434	0.1434	0.1434	0.1434
		DS	0.0008	0.0009	0.0610	0.0008	0.0610
		IPTW KM	0.0085	0.0085	0.0085	0.0740	0.0740
		IPTW CH	-0.0024	-0.0026	-0.0024	0.0633	0.0633
		Matching	0.0072	0.0096	0.0072	0.0764	0.0764
		EL	-0.0001	-0.0004	-0.0001	0.0792	0.0792
		AIPTW	0.0073	0.0066	0.0073	0.0053	0.0738

Methods for deriving survival curves: KM, Standard Kaplan-Meier based approach (unadjusted); DS, Direct Standardization via a Cox model (G-formula); IPTW KM, Xie and Liu's approach; IPTW CH, Cole and Hernan's approach; Matching, Propensity score matching; EL, Empirical Likelihood approach; AIPTW, Augmented Inverse Probability of Treatment Weighting approach.

Case	Outcome model	Treatment model
1	Correct	Correct
2	Included extra variables, X_3 and X_6	Included extra variables, X_1 and X_4
3	Failed to include X_2	Correct
4	Correct	Failed to include X_2
5	Failed to include X_2	Failed to include X_2

Table B6: Square-root of Mean Squared Error (rMSE) for AH in Group 1

Scenario	Censoring	Method	Case 1	2	3	4	5
1	A	KM	0.1751	0.1751	0.1751	0.1751	0.1751
		DS	0.0899	0.0919	0.1056	0.0899	0.1056
		IPTW KM	0.1429	0.1427	0.1429	0.1501	0.1501
		IPTW CH	0.1403	0.1399	0.1403	0.1456	0.1456
		Matching	0.1636	0.1679	0.1636	0.1748	0.1748
		EL	0.1750	0.1600	0.1750	0.1664	0.1664
		AIPTW	0.1243	0.1252	0.1248	0.1174	0.1347
1	B	KM	0.1767	0.1767	0.1767	0.1767	0.1767
		DS	0.0907	0.0928	0.1064	0.0907	0.1064
		IPTW KM	0.1455	0.1452	0.1455	0.1520	0.1520
		IPTW CH	0.1428	0.1423	0.1428	0.1474	0.1474
		Matching	0.1661	0.1707	0.1661	0.1766	0.1766
		EL	0.1777	0.1616	0.1777	0.1673	0.1673
		AIPTW	0.1281	0.1291	0.1286	0.1210	0.1376
2	A	KM	0.2807	0.2807	0.2807	0.2807	0.2807
		DS	0.1433	0.1463	0.1742	0.1433	0.1742
		IPTW KM	0.2232	0.2214	0.2232	0.2404	0.2404
		IPTW CH	0.2179	0.2157	0.2179	0.2313	0.2313
		Matching	0.2594	0.2641	0.2594	0.2866	0.2866
		EL	0.2748	0.2452	0.2748	0.2682	0.2682
		AIPTW	0.1932	0.1944	0.1942	0.1810	0.2177
2	B	KM	0.2830	0.2830	0.2830	0.2830	0.2830
		DS	0.1446	0.1479	0.1756	0.1446	0.1756
		IPTW KM	0.2262	0.2249	0.2262	0.2430	0.2430
		IPTW CH	0.2207	0.2190	0.2207	0.2336	0.2336
		Matching	0.2630	0.2689	0.2630	0.2891	0.2891
		EL	0.2785	0.2499	0.2785	0.2707	0.2707
		AIPTW	0.1971	0.1985	0.1979	0.1843	0.2207

Methods for deriving survival curves: KM, Standard Kaplan-Meier based approach (unadjusted); DS, Direct Standardization via a Cox model (G-formula); IPTW KM, Xie and Liu's approach; IPTW CH, Cole and Hernan's approach; Matching, Propensity score matching; EL, Empirical Likelihood approach; AIPTW, Augmented Inverse Probability of Treatment Weighting approach.

Case	Outcome model	Treatment model
1	Correct	Correct
2	Included extra variables, X_3 and X_6	Included extra variables, X_1 and X_4
3	Failed to include X_2	Correct
4	Correct	Failed to include X_2
5	Failed to include X_2	Failed to include X_2

Table B7: Coverage Probability of 0.95 Confidence Interval for AH in Group 1

			Case				
Scenario	Censoring	Method	1	2	3	4	5
1	A	KM	0.873	0.873	0.873	0.873	0.873
		DS	0.951	0.953	0.939	0.951	0.939
		IPTW KM	0.948	0.954	0.948	0.949	0.949
		IPTW CH	0.946	0.949	0.946	0.950	0.950
		Matching	0.944	0.951	0.944	0.937	0.937
		EL	0.945	0.954	0.945	0.939	0.939
		AIPTW	0.946	0.947	0.947	0.951	0.943
1	B	KM	0.873	0.873	0.873	0.873	0.873
		DS	0.948	0.950	0.942	0.948	0.942
		IPTW KM	0.946	0.949	0.946	0.953	0.953
		IPTW CH	0.943	0.946	0.943	0.955	0.955
		Matching	0.946	0.948	0.946	0.945	0.945
		EL	0.943	0.953	0.943	0.946	0.946
		AIPTW	0.948	0.950	0.949	0.944	0.948
2	A	KM	0.906	0.906	0.906	0.906	0.906
		DS	0.956	0.958	0.945	0.956	0.945
		IPTW KM	0.947	0.951	0.947	0.957	0.957
		IPTW CH	0.942	0.948	0.942	0.961	0.961
		Matching	0.948	0.950	0.948	0.935	0.935
		EL	0.949	0.958	0.949	0.951	0.951
		AIPTW	0.952	0.952	0.952	0.952	0.957
2	B	KM	0.903	0.903	0.903	0.903	0.903
		DS	0.956	0.956	0.948	0.956	0.948
		IPTW KM	0.946	0.951	0.946	0.960	0.960
		IPTW CH	0.942	0.944	0.942	0.962	0.962
		Matching	0.948	0.950	0.948	0.940	0.940
		EL	0.946	0.953	0.946	0.953	0.953
		AIPTW	0.950	0.951	0.952	0.954	0.956

Methods for deriving survival curves: KM, Standard Kaplan-Meier based approach (unadjusted); DS, Direct Standardization via a Cox model (G-formula); IPTW KM, Xie and Liu's approach; IPTW CH, Cole and Hernan's approach; Matching, Propensity score matching; EL, Empirical Likelihood approach; AIPTW, Augmented Inverse Probability of Treatment Weighting approach.

Case	Outcome model	Treatment model
1	Correct	Correct
2	Included extra variables, X_3 and X_6	Included extra variables, X_1 and X_4
3	Failed to include X_2	Correct
4	Correct	Failed to include X_2
5	Failed to include X_2	Failed to include X_2

Table B8: Median Length of 0.95 Confidence Interval for AH in Group 1

			Case				
Scenario	Censoring	Method	1	2	3	4	5
1	A	KM	0.4977	0.4977	0.4977	0.4977	0.4977
		DS	0.3556	0.3627	0.3709	0.3556	0.3709
		IPTW KM	0.5423	0.5360	0.5423	0.5341	0.5341
		IPTW CH	0.5345	0.5274	0.5345	0.5274	0.5274
		Matching	0.6309	0.6403	0.6309	0.6192	0.6192
		EL	0.6250	0.6068	0.6250	0.5740	0.5740
		AIPTW	0.4711	0.4768	0.4746	0.4530	0.4729
1	B	KM	0.5063	0.5063	0.5063	0.5063	0.5063
		DS	0.3624	0.3693	0.3771	0.3624	0.3771
		IPTW KM	0.5517	0.5452	0.5517	0.5439	0.5439
		IPTW CH	0.5431	0.5363	0.5431	0.5367	0.5367
		Matching	0.6430	0.6518	0.6430	0.6293	0.6293
		EL	0.6361	0.6174	0.6361	0.5850	0.5850
		AIPTW	0.4901	0.4939	0.4910	0.4705	0.4903
2	A	KM	0.8089	0.8089	0.8089	0.8089	0.8089
		DS	0.5718	0.5833	0.6067	0.5718	0.6067
		IPTW KM	0.8632	0.8499	0.8632	0.8601	0.8601
		IPTW CH	0.8458	0.8316	0.8458	0.8446	0.8446
		Matching	1.0048	1.0208	1.0048	1.0019	1.0019
		EL	0.9964	0.9575	0.9964	0.9251	0.9251
		AIPTW	0.7426	0.7517	0.7469	0.7055	0.7557
2	B	KM	0.8167	0.8167	0.8167	0.8167	0.8167
		DS	0.5774	0.5902	0.6126	0.5774	0.6126
		IPTW KM	0.8725	0.8603	0.8725	0.8718	0.8718
		IPTW CH	0.8552	0.8415	0.8552	0.8565	0.8565
		Matching	1.0169	1.0322	1.0169	1.0145	1.0145
		EL	1.0041	0.9702	1.0041	0.9357	0.9357
		AIPTW	0.7585	0.7712	0.7649	0.7216	0.7728

Methods for deriving survival curves: KM, Standard Kaplan-Meier based approach (unadjusted); DS, Direct Standardization via a Cox model (G-formula); IPTW KM, Xie and Liu's approach; IPTW CH, Cole and Hernan's approach; Matching, Propensity score matching; EL, Empirical Likelihood approach; AIPTW, Augmented Inverse Probability of Treatment Weighting approach.

Case	Outcome model	Treatment model
1	Correct	Correct
2	Included extra variables, X_3 and X_6	Included extra variables, X_1 and X_4
3	Failed to include X_2	Correct
4	Correct	Failed to include X_2
5	Failed to include X_2	Failed to include X_2

Table B9: Mean Relative Bias for log(RAH)

Scenario	Censoring	Method	Case 1	2	3	4	5
1	A	KM	-0.2960	-0.2960	-0.2960	-0.2960	-0.2960
		DS	-0.0001	0.0006	-0.1322	-0.0001	-0.1322
		IPTW KM	0.0044	0.0032	0.0044	-0.1405	-0.1405
		IPTW CH	0.0080	0.0070	0.0080	-0.1368	-0.1368
		Matching	0.0184	0.0093	0.0184	-0.1312	-0.1312
		EL	0.0118	0.0181	0.0118	-0.1446	-0.1446
		AIPTW	-0.0001	0.0016	-0.0005	0.0012	-0.1453
1	B	KM	-0.2962	-0.2962	-0.2962	-0.2962	-0.2962
		DS	-0.0003	0.0003	-0.1321	-0.0003	-0.1321
		IPTW KM	0.0052	0.0042	0.0052	-0.1395	-0.1395
		IPTW CH	0.0090	0.0081	0.0090	-0.1357	-0.1357
		Matching	0.0190	0.0098	0.0190	-0.1309	-0.1309
		EL	0.0113	0.0191	0.0113	-0.1431	-0.1431
		AIPTW	0.0005	0.0022	0.0001	0.0019	-0.1444
2	A	KM	-0.2705	-0.2705	-0.2705	-0.2705	-0.2705
		DS	-0.0018	-0.0015	-0.1302	-0.0018	-0.1302
		IPTW KM	0.0051	0.0033	0.0051	-0.1375	-0.1375
		IPTW CH	0.0071	0.0055	0.0071	-0.1351	-0.1351
		Matching	0.0119	0.0085	0.0119	-0.1302	-0.1302
		EL	0.0095	0.0136	0.0095	-0.1432	-0.1432
		AIPTW	0.0013	0.0022	0.0008	0.0022	-0.1420
2	B	KM	-0.2714	-0.2714	-0.2714	-0.2714	-0.2714
		DS	-0.0023	-0.0022	-0.1307	-0.0023	-0.1307
		IPTW KM	0.0038	0.0022	0.0038	-0.1384	-0.1384
		IPTW CH	0.0060	0.0045	0.0060	-0.1359	-0.1359
		Matching	0.0107	0.0067	0.0107	-0.1310	-0.1310
		EL	0.0080	0.0118	0.0080	-0.1441	-0.1441
		AIPTW	0.0005	0.0016	-0.0000	0.0016	-0.1425

Methods for deriving survival curves: KM, Standard Kaplan-Meier based approach (unadjusted); DS, Direct Standardization via a Cox model (G-formula); IPTW KM, Xie and Liu's approach; IPTW CH, Cole and Hernan's approach; Matching, Propensity score matching; EL, Empirical Likelihood approach; AIPTW, Augmented Inverse Probability of Treatment Weighting approach.

Case	Outcome model	Treatment model
1	Correct	Correct
2	Included extra variables, X_3 and X_6	Included extra variables, X_1 and X_4
3	Failed to include X_2	Correct
4	Correct	Failed to include X_2
5	Failed to include X_2	Failed to include X_2

Table B10: Square-root of Mean Squared Error (rMSE) for log(RAH)

			Case				
Scenario	Censoring	Method	1	2	3	4	5
1	A	KM	0.2341	0.2341	0.2341	0.2341	0.2341
		DS	0.0934	0.0984	0.1244	0.0934	0.1244
		IPTW KM	0.1770	0.1724	0.1770	0.1880	0.1880
		IPTW CH	0.1757	0.1709	0.1757	0.1863	0.1863
		Matching	0.2032	0.2021	0.2032	0.2095	0.2095
		EL	0.3425	0.3015	0.3425	0.2067	0.2067
		AIPTW	0.1453	0.1468	0.1465	0.1385	0.1631
1	B	KM	0.2361	0.2361	0.2361	0.2361	0.2361
		DS	0.0943	0.0996	0.1252	0.0943	0.1252
		IPTW KM	0.1805	0.1763	0.1805	0.1905	0.1905
		IPTW CH	0.1792	0.1748	0.1792	0.1888	0.1888
		Matching	0.2067	0.2062	0.2067	0.2121	0.2121
		EL	0.3463	0.3044	0.3463	0.2084	0.2084
		AIPTW	0.1528	0.1545	0.1541	0.1462	0.1691
2	A	KM	0.2614	0.2614	0.2614	0.2614	0.2614
		DS	0.1094	0.1151	0.1484	0.1094	0.1484
		IPTW KM	0.1939	0.1869	0.1939	0.2118	0.2118
		IPTW CH	0.1922	0.1850	0.1922	0.2098	0.2098
		Matching	0.2254	0.2224	0.2254	0.2376	0.2376
		EL	0.3704	0.3170	0.3704	0.2319	0.2319
		AIPTW	0.1588	0.1600	0.1600	0.1512	0.1851
2	B	KM	0.2631	0.2631	0.2631	0.2631	0.2631
		DS	0.1102	0.1163	0.1492	0.1102	0.1492
		IPTW KM	0.1963	0.1898	0.1963	0.2138	0.2138
		IPTW CH	0.1945	0.1878	0.1945	0.2117	0.2117
		Matching	0.2279	0.2259	0.2279	0.2398	0.2398
		EL	0.3732	0.3193	0.3732	0.2335	0.2335
		AIPTW	0.1628	0.1643	0.1638	0.1551	0.1881

Methods for deriving survival curves: KM, Standard Kaplan-Meier based approach (unadjusted); DS, Direct Standardization via a Cox model (G-formula); IPTW KM, Xie and Liu's approach; IPTW CH, Cole and Hernan's approach; Matching, Propensity score matching; EL, Empirical Likelihood approach; AIPTW, Augmented Inverse Probability of Treatment Weighting approach.

Case	Outcome model	Treatment model
1	Correct	Correct
2	Included extra variables, X_3 and X_6	Included extra variables, X_1 and X_4
3	Failed to include X_2	Correct
4	Correct	Failed to include X_2
5	Failed to include X_2	Failed to include X_2

Table B11: Coverage Probability of 0.95 Confidence Interval for RAH

			Case				
Scenario	Censoring	Method	1	2	3	4	5
1	A	KM	0.747	0.747	0.747	0.747	0.747
		DS	0.953	0.952	0.847	0.953	0.847
		IPTW KM	0.947	0.947	0.947	0.906	0.906
		IPTW CH	0.947	0.947	0.947	0.906	0.906
		Matching	0.941	0.949	0.941	0.919	0.919
		EL	0.976	0.986	0.976	0.929	0.929
		AIPTW	0.952	0.951	0.954	0.944	0.886
1	B	KM	0.747	0.747	0.747	0.747	0.747
		DS	0.957	0.955	0.852	0.957	0.852
		IPTW KM	0.948	0.948	0.948	0.910	0.910
		IPTW CH	0.948	0.947	0.948	0.911	0.911
		Matching	0.944	0.949	0.944	0.919	0.919
		EL	0.974	0.985	0.974	0.935	0.935
		AIPTW	0.946	0.949	0.945	0.948	0.899
2	A	KM	0.764	0.764	0.764	0.764	0.764
		DS	0.949	0.952	0.853	0.949	0.853
		IPTW KM	0.950	0.948	0.950	0.909	0.909
		IPTW CH	0.951	0.948	0.951	0.908	0.908
		Matching	0.942	0.946	0.942	0.918	0.918
		EL	0.976	0.990	0.976	0.931	0.931
		AIPTW	0.949	0.950	0.949	0.951	0.892
2	B	KM	0.773	0.773	0.773	0.773	0.773
		DS	0.955	0.953	0.851	0.955	0.851
		IPTW KM	0.952	0.948	0.952	0.905	0.905
		IPTW CH	0.952	0.948	0.952	0.905	0.905
		Matching	0.941	0.946	0.941	0.918	0.918
		EL	0.976	0.989	0.976	0.932	0.932
		AIPTW	0.953	0.955	0.951	0.954	0.898

Methods for deriving survival curves: KM, Standard Kaplan-Meier based approach (unadjusted); DS, Direct Standardization via a Cox model (G-formula); IPTW KM, Xie and Liu's approach; IPTW CH, Cole and Hernan's approach; Matching, Propensity score matching; EL, Empirical Likelihood approach; AIPTW, Augmented Inverse Probability of Treatment Weighting approach.

Case	Outcome model	Treatment model
1	Correct	Correct
2	Included extra variables, X_3 and X_6	Included extra variables, X_1 and X_4
3	Failed to include X_2	Correct
4	Correct	Failed to include X_2
5	Failed to include X_2	Failed to include X_2

Table B12: Median Length of 0.95 Confidence Interval for log(RAH)

			Case				
Scenario	Censoring	Method	1	2	3	4	5
1	A	KM	0.5728	0.5728	0.5728	0.5728	0.5728
		DS	0.3641	0.3831	0.3653	0.3641	0.3653
		IPTW KM	0.6705	0.6467	0.6705	0.6400	0.6400
		IPTW CH	0.6670	0.6420	0.6670	0.6367	0.6367
		Matching	0.7835	0.7801	0.7835	0.7420	0.7420
		EL	1.2233	1.8063	1.2233	0.7165	0.7165
		AIPTW	0.5499	0.5547	0.5521	0.5316	0.5265
1	B	KM	0.5840	0.5840	0.5840	0.5840	0.5840
		DS	0.3723	0.3922	0.3735	0.3723	0.3735
		IPTW KM	0.6840	0.6598	0.6840	0.6518	0.6518
		IPTW CH	0.6799	0.6552	0.6799	0.6489	0.6489
		Matching	0.7980	0.7950	0.7980	0.7555	0.7555
		EL	1.2407	1.8240	1.2407	0.7271	0.7271
		AIPTW	0.5782	0.5848	0.5794	0.5604	0.5560
2	A	KM	0.6496	0.6496	0.6496	0.6496	0.6496
		DS	0.4270	0.4509	0.4338	0.4270	0.4338
		IPTW KM	0.7458	0.7117	0.7458	0.7126	0.7126
		IPTW CH	0.7392	0.7052	0.7392	0.7078	0.7078
		Matching	0.8696	0.8657	0.8696	0.8288	0.8288
		EL	1.3260	1.9308	1.3260	0.7919	0.7919
		AIPTW	0.6058	0.6112	0.6105	0.5845	0.5851
2	B	KM	0.6573	0.6573	0.6573	0.6573	0.6573
		DS	0.4333	0.4565	0.4402	0.4333	0.4402
		IPTW KM	0.7543	0.7206	0.7543	0.7215	0.7215
		IPTW CH	0.7478	0.7134	0.7478	0.7169	0.7169
		Matching	0.8793	0.8750	0.8793	0.8387	0.8387
		EL	1.3355	1.9363	1.3355	0.7998	0.7998
		AIPTW	0.6272	0.6339	0.6302	0.6058	0.6059

Methods for deriving survival curves: KM, Standard Kaplan-Meier based approach (unadjusted); DS, Direct Standardization via a Cox model (G-formula); IPTW KM, Xie and Liu's approach; IPTW CH, Cole and Hernan's approach; Matching, Propensity score matching; EL, Empirical Likelihood approach; AIPTW, Augmented Inverse Probability of Treatment Weighting approach.

Case	Outcome model	Treatment model
1	Correct	Correct
2	Included extra variables, X_3 and X_6	Included extra variables, X_1 and X_4
3	Failed to include X_2	Correct
4	Correct	Failed to include X_2
5	Failed to include X_2	Failed to include X_2

C Simulation Results with Censoring Pattern C

C.1 DAH

Table C1: Mean Relative Bias for DAH

			Case				
Scenario	Censoring	Method	1	2	3	4	5
1	C	KM	-0.2892	-0.2892	-0.2892	-0.2892	-0.2892
		DS	-0.0069	-0.0069	-0.1305	-0.0069	-0.1305
		IPTW KM	-0.0125	-0.0141	-0.0125	-0.1425	-0.1425
		IPTW CH	-0.0152	-0.0167	-0.0152	-0.1439	-0.1439
		Matching	-0.0043	-0.0139	-0.0043	-0.1387	-0.1387
		EL	-0.0009	-0.0009	-0.0009	-0.1501	-0.1501
		AIPTW	-0.0133	-0.0128	-0.0140	0.0384	-0.1007
2	C	KM	-0.2630	-0.2630	-0.2630	-0.2630	-0.2630
		DS	-0.0059	-0.0059	-0.1309	-0.0059	-0.1309
		IPTW KM	-0.0055	-0.0078	-0.0055	-0.1377	-0.1377
		IPTW CH	-0.0123	-0.0145	-0.0123	-0.1425	-0.1425
		Matching	-0.0021	-0.0085	-0.0021	-0.1332	-0.1332
		EL	0.0027	0.0038	0.0027	-0.1463	-0.1463
		AIPTW	-0.0033	-0.0033	-0.0041	0.0302	-0.1093

Methods for deriving survival curves: KM, Standard Kaplan-Meier based approach (unadjusted); DS, Direct Standardization via a Cox model (G-formula); IPTW KM, Xie and Liu's approach; IPTW CH, Cole and Hernan's approach; Matching, Propensity score matching; EL, Empirical Likelihood approach; AIPTW, Augmented Inverse Probability of Treatment Weighting approach.

Case	Outcome model	Treatment model
1	Correct	Correct
2	Included extra variables, X_3 and X_6	Included extra variables, X_1 and X_4
3	Failed to include X_2	Correct
4	Correct	Failed to include X_2
5	Failed to include X_2	Failed to include X_2

Table C2: Square-root of Mean Squared Error (rMSE) for DAH

			Case				
Scenario	Censoring	Method	1	2	3	4	5
1	C	KM	0.2942	0.2942	0.2942	0.2942	0.2942
		DS	0.1156	0.1217	0.1555	0.1156	0.1555
		IPTW KM	0.2066	0.2001	0.2066	0.2303	0.2303
		IPTW CH	0.2037	0.1970	0.2037	0.2289	0.2289
		Matching	0.2349	0.2322	0.2349	0.2530	0.2530
		EL	0.2571	0.2356	0.2571	0.2497	0.2497
		AIPTW	0.1769	0.1778	0.1779	0.1715	0.1857
2	C	KM	0.5207	0.5207	0.5207	0.5207	0.5207
		DS	0.2351	0.2458	0.3058	0.2351	0.3058
		IPTW KM	0.3733	0.3588	0.3733	0.4188	0.4188
		IPTW CH	0.3671	0.3524	0.3671	0.4179	0.4179
		Matching	0.4335	0.4223	0.4335	0.4645	0.4645
		EL	0.4563	0.4168	0.4563	0.4503	0.4503
		AIPTW	0.3190	0.3201	0.3205	0.3119	0.3449

Methods for deriving survival curves: KM, Standard Kaplan-Meier based approach (unadjusted); DS, Direct Standardization via a Cox model (G-formula); IPTW KM, Xie and Liu's approach; IPTW CH, Cole and Hernan's approach; Matching, Propensity score matching; EL, Empirical Likelihood approach; AIPTW, Augmented Inverse Probability of Treatment Weighting approach.

Case	Outcome model	Treatment model
1	Correct	Correct
2	Included extra variables, X_3 and X_6	Included extra variables, X_1 and X_4
3	Failed to include X_2	Correct
4	Correct	Failed to include X_2
5	Failed to include X_2	Failed to include X_2

Table C3: Coverage Probability of 0.95 Confidence Interval for DAH

			Case				
Scenario	Censoring	Method	1	2	3	4	5
1	C	KM	0.757	0.757	0.757	0.757	0.757
		DS	0.950	0.950	0.859	0.950	0.859
		IPTW KM	0.953	0.953	0.953	0.909	0.909
		IPTW CH	0.953	0.950	0.953	0.907	0.907
		Matching	0.950	0.951	0.950	0.927	0.927
		EL	0.962	0.972	0.962	0.918	0.918
		AIPTW	0.956	0.956	0.956	0.949	0.927
2	C	KM	0.772	0.772	0.772	0.772	0.772
		DS	0.955	0.958	0.845	0.955	0.845
		IPTW KM	0.957	0.956	0.957	0.910	0.910
		IPTW CH	0.955	0.957	0.955	0.908	0.908
		Matching	0.957	0.957	0.957	0.921	0.921
		EL	0.965	0.974	0.965	0.917	0.917
		AIPTW	0.960	0.964	0.964	0.961	0.929

Methods for deriving survival curves: KM, Standard Kaplan-Meier based approach (unadjusted); DS, Direct Standardization via a Cox model (G-formula); IPTW KM, Xie and Liu's approach; IPTW CH, Cole and Hernan's approach; Matching, Propensity score matching; EL, Empirical Likelihood approach; AIPTW, Augmented Inverse Probability of Treatment Weighting approach.

Case	Outcome model	Treatment model
1	Correct	Correct
2	Included extra variables, X_3 and X_6	Included extra variables, X_1 and X_4
3	Failed to include X_2	Correct
4	Correct	Failed to include X_2
5	Failed to include X_2	Failed to include X_2

Table C4: Median Length of 0.95 Confidence Interval for DAH

			Case				
Scenario	Censoring	Method	1	2	3	4	5
1	C	KM	0.7206	0.7206	0.7206	0.7206	0.7206
		DS	0.4538	0.4770	0.4550	0.4538	0.4550
		IPTW KM	0.7886	0.7557	0.7886	0.7702	0.7702
		IPTW CH	0.7780	0.7436	0.7780	0.7614	0.7614
		Matching	0.9088	0.9060	0.9088	0.8851	0.8851
		EL	0.9401	0.9901	0.9401	0.8307	0.8307
		AIPTW	0.6784	0.6842	0.6780	0.6565	0.6536
2	C	KM	1.3388	1.3388	1.3388	1.3388	1.3388
		DS	0.9345	0.9803	0.9215	0.9345	0.9215
		IPTW KM	1.4807	1.4100	1.4807	1.4355	1.4355
		IPTW CH	1.4521	1.3809	1.4521	1.4133	1.4133
		Matching	1.6996	1.6800	1.6996	1.6506	1.6506
		EL	1.7252	1.7938	1.7252	1.5243	1.5243
		AIPTW	1.2685	1.2744	1.2713	1.2388	1.2244

Methods for deriving survival curves: KM, Standard Kaplan-Meier based approach (unadjusted); DS, Direct Standardization via a Cox model (G-formula); IPTW KM, Xie and Liu's approach; IPTW CH, Cole and Hernan's approach; Matching, Propensity score matching; EL, Empirical Likelihood approach; AIPTW, Augmented Inverse Probability of Treatment Weighting approach.

Case	Outcome model	Treatment model
1	Correct	Correct
2	Included extra variables, X_3 and X_6	Included extra variables, X_1 and X_4
3	Failed to include X_2	Correct
4	Correct	Failed to include X_2
5	Failed to include X_2	Failed to include X_2

C.2 AH in each group and RAH

Table C5: Mean Relative Bias for AH in Group 0

			Case				
Scenario	Censoring	Method	1	2	3	4	5
1	C	KM	-0.0699	-0.0699	-0.0699	-0.0699	-0.0699
		DS	-0.0037	-0.0038	-0.0412	-0.0037	-0.0412
		IPTW KM	-0.0099	-0.0107	-0.0099	-0.0398	-0.0398
		IPTW CH	-0.0158	-0.0167	-0.0158	-0.0449	-0.0449
		Matching	-0.0097	-0.0117	-0.0097	-0.0376	-0.0376
		EL	-0.0104	-0.0102	-0.0104	-0.0419	-0.0419
		AIPTW	-0.0081	-0.0084	-0.0084	0.0048	-0.0279
2	C	KM	-0.0756	-0.0756	-0.0756	-0.0756	-0.0756
		DS	-0.0025	-0.0025	-0.0462	-0.0025	-0.0462
		IPTW KM	-0.0028	-0.0040	-0.0028	-0.0423	-0.0423
		IPTW CH	-0.0115	-0.0127	-0.0115	-0.0498	-0.0498
		Matching	-0.0022	-0.0043	-0.0022	-0.0385	-0.0385
		EL	-0.0028	-0.0026	-0.0028	-0.0446	-0.0446
		AIPTW	-0.0009	-0.0012	-0.0014	0.0088	-0.0330

Methods for deriving survival curves: KM, Standard Kaplan-Meier based approach (unadjusted); DS, Direct Standardization via a Cox model (G-formula); IPTW KM, Xie and Liu's approach; IPTW CH, Cole and Hernan's approach; Matching, Propensity score matching; EL, Empirical Likelihood approach; AIPTW, Augmented Inverse Probability of Treatment Weighting approach.

Case	Outcome model	Treatment model
1	Correct	Correct
2	Included extra variables, X_3 and X_6	Included extra variables, X_1 and X_4
3	Failed to include X_2	Correct
4	Correct	Failed to include X_2
5	Failed to include X_2	Failed to include X_2

Table C6: Square-root of Mean Squared Error (rMSE) for AH in Group 0

			Case				
Scenario	Censoring	Method	1	2	3	4	5
1	C	KM	0.1747	0.1747	0.1747	0.1747	0.1747
		DS	0.1142	0.1158	0.1317	0.1142	0.1317
		IPTW KM	0.1446	0.1417	0.1446	0.1544	0.1544
		IPTW CH	0.1447	0.1419	0.1447	0.1576	0.1576
		Matching	0.1645	0.1604	0.1645	0.1684	0.1684
		EL	0.1768	0.1658	0.1768	0.1603	0.1603
		AIPTW	0.1375	0.1376	0.1386	0.1319	0.1369
2	C	KM	0.3388	0.3388	0.3388	0.3388	0.3388
		DS	0.2418	0.2456	0.2671	0.2418	0.2671
		IPTW KM	0.2997	0.2932	0.2997	0.3101	0.3101
		IPTW CH	0.2969	0.2907	0.2969	0.3159	0.3159
		Matching	0.3410	0.3337	0.3410	0.3406	0.3406
		EL	0.3526	0.3347	0.3526	0.3201	0.3201
		AIPTW	0.2815	0.2817	0.2829	0.2778	0.2805

Methods for deriving survival curves: KM, Standard Kaplan-Meier based approach (unadjusted); DS, Direct Standardization via a Cox model (G-formula); IPTW KM, Xie and Liu's approach; IPTW CH, Cole and Hernan's approach; Matching, Propensity score matching; EL, Empirical Likelihood approach; AIPTW, Augmented Inverse Probability of Treatment Weighting approach.

Case	Outcome model	Treatment model
1	Correct	Correct
2	Included extra variables, X_3 and X_6	Included extra variables, X_1 and X_4
3	Failed to include X_2	Correct
4	Correct	Failed to include X_2
5	Failed to include X_2	Failed to include X_2

Table C7: Coverage Probability of 0.95 Confidence Interval for AH in Group 0

			Case				
Scenario	Censoring	Method	1	2	3	4	5
1	C	KM	0.831	0.831	0.831	0.831	0.831
		DS	0.953	0.952	0.903	0.953	0.903
		IPTW KM	0.949	0.951	0.949	0.913	0.913
		IPTW CH	0.941	0.943	0.941	0.902	0.902
		Matching	0.954	0.951	0.954	0.924	0.924
		EL	0.966	0.980	0.966	0.921	0.921
		AIPTW	0.951	0.953	0.950	0.955	0.939
2	C	KM	0.839	0.839	0.839	0.839	0.839
		DS	0.955	0.954	0.890	0.955	0.890
		IPTW KM	0.955	0.951	0.955	0.910	0.910
		IPTW CH	0.945	0.945	0.945	0.897	0.897
		Matching	0.953	0.956	0.953	0.926	0.926
		EL	0.963	0.976	0.963	0.919	0.919
		AIPTW	0.952	0.955	0.954	0.957	0.932

Methods for deriving survival curves: KM, Standard Kaplan-Meier based approach (unadjusted); DS, Direct Standardization via a Cox model (G-formula); IPTW KM, Xie and Liu's approach; IPTW CH, Cole and Hernan's approach; Matching, Propensity score matching; EL, Empirical Likelihood approach; AIPTW, Augmented Inverse Probability of Treatment Weighting approach.

Case	Outcome model	Treatment model
1	Correct	Correct
2	Included extra variables, X_3 and X_6	Included extra variables, X_1 and X_4
3	Failed to include X_2	Correct
4	Correct	Failed to include X_2
5	Failed to include X_2	Failed to include X_2

Table C8: Median Length of 0.95 Confidence Interval for AH in Group 0

			Case				
Scenario	Censoring	Method	1	2	3	4	5
1	C	KM	0.5120	0.5120	0.5120	0.5120	0.5120
		DS	0.4627	0.4692	0.4514	0.4627	0.4514
		IPTW KM	0.5716	0.5613	0.5716	0.5515	0.5515
		IPTW CH	0.5660	0.5555	0.5660	0.5469	0.5469
		Matching	0.6472	0.6457	0.6472	0.6270	0.6270
		EL	0.6776	0.7724	0.6776	0.5814	0.5814
		AIPTW	0.5497	0.5514	0.5500	0.5330	0.5191
2	C	KM	1.0511	1.0511	1.0511	1.0511	1.0511
		DS	0.9769	0.9902	0.9399	0.9769	0.9399
		IPTW KM	1.2001	1.1716	1.2001	1.1399	1.1399
		IPTW CH	1.1812	1.1521	1.1812	1.1244	1.1244
		Matching	1.3560	1.3526	1.3560	1.2997	1.2997
		EL	1.3713	1.5128	1.3713	1.1913	1.1913
		AIPTW	1.1343	1.1381	1.1371	1.1153	1.0668

Methods for deriving survival curves: KM, Standard Kaplan-Meier based approach (unadjusted); DS, Direct Standardization via a Cox model (G-formula); IPTW KM, Xie and Liu's approach; IPTW CH, Cole and Hernan's approach; Matching, Propensity score matching; EL, Empirical Likelihood approach; AIPTW, Augmented Inverse Probability of Treatment Weighting approach.

Case	Outcome model	Treatment model
1	Correct	Correct
2	Included extra variables, X_3 and X_6	Included extra variables, X_1 and X_4
3	Failed to include X_2	Correct
4	Correct	Failed to include X_2
5	Failed to include X_2	Failed to include X_2

Table C9: Mean Relative Bias for AH in Group 1

Scenario	Censoring	Method	Case 1	2	3	4	5
1	C	KM	0.1168	0.1168	0.1168	0.1168	0.1168
		DS	-0.0009	-0.0011	0.0347	-0.0009	0.0347
		IPTW KM	-0.0076	-0.0078	-0.0076	0.0477	0.0477
		IPTW CH	-0.0162	-0.0166	-0.0162	0.0394	0.0394
		Matching	-0.0143	-0.0099	-0.0143	0.0484	0.0484
		EL	-0.0185	-0.0181	-0.0185	0.0503	0.0503
		AIPTW	-0.0037	-0.0046	-0.0037	-0.0238	0.0340
2	C	KM	0.1342	0.1342	0.1342	0.1342	0.1342
		DS	0.0013	0.0013	0.0486	0.0013	0.0486
		IPTW KM	0.0003	0.0003	0.0003	0.0644	0.0644
		IPTW CH	-0.0105	-0.0107	-0.0105	0.0539	0.0539
		Matching	-0.0023	0.0004	-0.0023	0.0675	0.0675
		EL	-0.0089	-0.0097	-0.0089	0.0692	0.0692
		AIPTW	0.0017	0.0012	0.0017	-0.0150	0.0524

Methods for deriving survival curves: KM, Standard Kaplan-Meier based approach (unadjusted); DS, Direct Standardization via a Cox model (G-formula); IPTW KM, Xie and Liu's approach; IPTW CH, Cole and Hernan's approach; Matching, Propensity score matching; EL, Empirical Likelihood approach; AIPTW, Augmented Inverse Probability of Treatment Weighting approach.

Case	Outcome model	Treatment model
1	Correct	Correct
2	Included extra variables, X_3 and X_6	Included extra variables, X_1 and X_4
3	Failed to include X_2	Correct
4	Correct	Failed to include X_2
5	Failed to include X_2	Failed to include X_2

Table C10: Square-root of Mean Squared Error (rMSE) for AH in Group 1

			Case				
Scenario	Censoring	Method	1	2	3	4	5
1	C	KM	0.1691	0.1691	0.1691	0.1691	0.1691
		DS	0.0903	0.0923	0.0993	0.0903	0.0993
		IPTW KM	0.1455	0.1452	0.1455	0.1478	0.1478
		IPTW CH	0.1434	0.1429	0.1434	0.1439	0.1439
		Matching	0.1664	0.1704	0.1664	0.1719	0.1719
		EL	0.1779	0.1623	0.1779	0.1641	0.1641
		AIPTW	0.1259	0.1269	0.1263	0.1226	0.1287
2	C	KM	0.2736	0.2736	0.2736	0.2736	0.2736
		DS	0.1443	0.1474	0.1669	0.1443	0.1669
		IPTW KM	0.2243	0.2226	0.2243	0.2360	0.2360
		IPTW CH	0.2197	0.2176	0.2197	0.2274	0.2274
		Matching	0.2610	0.2652	0.2610	0.2823	0.2823
		EL	0.2760	0.2465	0.2760	0.2630	0.2630
		AIPTW	0.1928	0.1941	0.1934	0.1822	0.2046

Methods for deriving survival curves: KM, Standard Kaplan-Meier based approach (unadjusted); DS, Direct Standardization via a Cox model (G-formula); IPTW KM, Xie and Liu's approach; IPTW CH, Cole and Hernan's approach; Matching, Propensity score matching; EL, Empirical Likelihood approach; AIPTW, Augmented Inverse Probability of Treatment Weighting approach.

Case	Outcome model	Treatment model
1	Correct	Correct
2	Included extra variables, X_3 and X_6	Included extra variables, X_1 and X_4
3	Failed to include X_2	Correct
4	Correct	Failed to include X_2
5	Failed to include X_2	Failed to include X_2

Table C11: Coverage Probability of 0.95 Confidence Interval for AH in Group 1

			Case				
Scenario	Censoring	Method	1	2	3	4	5
1	C	KM	0.894	0.894	0.894	0.894	0.894
		DS	0.956	0.953	0.944	0.956	0.944
		IPTW KM	0.940	0.944	0.940	0.952	0.952
		IPTW CH	0.937	0.939	0.937	0.956	0.956
		Matching	0.940	0.943	0.940	0.941	0.941
		EL	0.937	0.946	0.937	0.946	0.946
		AIPTW	0.939	0.940	0.938	0.929	0.950
2	C	KM	0.915	0.915	0.915	0.915	0.915
		DS	0.954	0.957	0.953	0.954	0.953
		IPTW KM	0.943	0.950	0.943	0.964	0.964
		IPTW CH	0.941	0.942	0.941	0.963	0.963
		Matching	0.949	0.944	0.949	0.945	0.945
		EL	0.944	0.946	0.944	0.957	0.957
		AIPTW	0.948	0.947	0.947	0.939	0.965

Methods for deriving survival curves: KM, Standard Kaplan-Meier based approach (unadjusted); DS, Direct Standardization via a Cox model (G-formula); IPTW KM, Xie and Liu's approach; IPTW CH, Cole and Hernan's approach; Matching, Propensity score matching; EL, Empirical Likelihood approach; AIPTW, Augmented Inverse Probability of Treatment Weighting approach.

Case	Outcome model	Treatment model
1	Correct	Correct
2	Included extra variables, X_3 and X_6	Included extra variables, X_1 and X_4
3	Failed to include X_2	Correct
4	Correct	Failed to include X_2
5	Failed to include X_2	Failed to include X_2

Table C12: Median Length of 0.95 Confidence Interval for AH in Group 1

			Case				
Scenario	Censoring	Method	1	2	3	4	5
1	C	KM	0.5021	0.5021	0.5021	0.5021	0.5021
		DS	0.3585	0.3665	0.3722	0.3585	0.3722
		IPTW KM	0.5487	0.5419	0.5487	0.5381	0.5381
		IPTW CH	0.5404	0.5321	0.5404	0.5315	0.5315
		Matching	0.6376	0.6471	0.6376	0.6255	0.6255
		EL	0.6328	0.6147	0.6328	0.5790	0.5790
		AIPTW	0.4754	0.4787	0.4762	0.4598	0.4767
2	C	KM	0.8111	0.8111	0.8111	0.8111	0.8111
		DS	0.5742	0.5869	0.6046	0.5742	0.6046
		IPTW KM	0.8674	0.8551	0.8674	0.8629	0.8629
		IPTW CH	0.8499	0.8373	0.8499	0.8488	0.8488
		Matching	1.0097	1.0262	1.0097	1.0061	1.0061
		EL	0.9997	0.9632	0.9997	0.9299	0.9299
		AIPTW	0.7431	0.7523	0.7464	0.7089	0.7547

Methods for deriving survival curves: KM, Standard Kaplan-Meier based approach (unadjusted); DS, Direct Standardization via a Cox model (G-formula); IPTW KM, Xie and Liu's approach; IPTW CH, Cole and Hernan's approach; Matching, Propensity score matching; EL, Empirical Likelihood approach; AIPTW, Augmented Inverse Probability of Treatment Weighting approach.

Case	Outcome model	Treatment model
1	Correct	Correct
2	Included extra variables, X_3 and X_6	Included extra variables, X_1 and X_4
3	Failed to include X_2	Correct
4	Correct	Failed to include X_2
5	Failed to include X_2	Failed to include X_2

Table C13: Mean Relative Bias for log(RAH)

			Case				
Scenario	Censoring	Method	1	2	3	4	5
1	C	KM	-0.2895	-0.2895	-0.2895	-0.2895	-0.2895
		DS	-0.0006	-0.0002	-0.1198	-0.0006	-0.1198
		IPTW KM	0.0106	0.0096	0.0106	-0.1301	-0.1301
		IPTW CH	0.0147	0.0139	0.0147	-0.1261	-0.1261
		Matching	0.0265	0.0175	0.0265	-0.1226	-0.1226
		EL	0.0192	0.0235	0.0192	-0.1338	-0.1338
		AIPTW	0.0025	0.0039	0.0021	0.0565	-0.0913
2	C	KM	-0.2671	-0.2671	-0.2671	-0.2671	-0.2671
		DS	-0.0024	-0.0022	-0.1233	-0.0024	-0.1233
		IPTW KM	0.0067	0.0050	0.0067	-0.1326	-0.1326
		IPTW CH	0.0092	0.0076	0.0092	-0.1299	-0.1299
		Matching	0.0150	0.0099	0.0150	-0.1259	-0.1259
		EL	0.0116	0.0157	0.0116	-0.1382	-0.1382
		AIPTW	0.0034	0.0040	0.0029	0.0380	-0.1067

Methods for deriving survival curves: KM, Standard Kaplan-Meier based approach (unadjusted); DS, Direct Standardization via a Cox model (G-formula); IPTW KM, Xie and Liu's approach; IPTW CH, Cole and Hernan's approach; Matching, Propensity score matching; EL, Empirical Likelihood approach; AIPTW, Augmented Inverse Probability of Treatment Weighting approach.

Case	Outcome model	Treatment model
1	Correct	Correct
2	Included extra variables, X_3 and X_6	Included extra variables, X_1 and X_4
3	Failed to include X_2	Correct
4	Correct	Failed to include X_2
5	Failed to include X_2	Failed to include X_2

Table C14: Square-root of Mean Squared Error (rMSE) for log(RAH)

			Case				
Scenario	Censoring	Method	1	2	3	4	5
1	C	KM	0.2333	0.2333	0.2333	0.2333	0.2333
		DS	0.0944	0.0997	0.1210	0.0944	0.1210
		IPTW KM	0.1807	0.1760	0.1807	0.1883	0.1883
		IPTW CH	0.1794	0.1746	0.1794	0.1865	0.1865
		Matching	0.2071	0.2075	0.2071	0.2099	0.2099
		EL	0.3432	0.2988	0.3432	0.2078	0.2078
		AIPTW	0.1514	0.1528	0.1525	0.1509	0.1542
2	C	KM	0.2596	0.2596	0.2596	0.2596	0.2596
		DS	0.1097	0.1156	0.1454	0.1097	0.1454
		IPTW KM	0.1940	0.1873	0.1940	0.2097	0.2097
		IPTW CH	0.1923	0.1854	0.1923	0.2077	0.2077
		Matching	0.2267	0.2246	0.2267	0.2362	0.2362
		EL	0.3705	0.3164	0.3705	0.2308	0.2308
		AIPTW	0.1602	0.1613	0.1610	0.1563	0.1716

Methods for deriving survival curves: KM, Standard Kaplan-Meier based approach (unadjusted); DS, Direct Standardization via a Cox model (G-formula); IPTW KM, Xie and Liu's approach; IPTW CH, Cole and Hernan's approach; Matching, Propensity score matching; EL, Empirical Likelihood approach; AIPTW, Augmented Inverse Probability of Treatment Weighting approach.

Case	Outcome model	Treatment model
1	Correct	Correct
2	Included extra variables, X_3 and X_6	Included extra variables, X_1 and X_4
3	Failed to include X_2	Correct
4	Correct	Failed to include X_2
5	Failed to include X_2	Failed to include X_2

Table C15: Coverage Probability of 0.95 Confidence Interval for RAH

			Case				
Scenario	Censoring	Method	1	2	3	4	5
1	C	KM	0.752	0.752	0.752	0.752	0.752
		DS	0.953	0.949	0.867	0.953	0.867
		IPTW KM	0.951	0.947	0.951	0.909	0.909
		IPTW CH	0.951	0.948	0.951	0.910	0.910
		Matching	0.944	0.949	0.944	0.921	0.921
		EL	0.977	0.986	0.977	0.934	0.934
		AIPTW	0.944	0.950	0.951	0.947	0.930
2	C	KM	0.769	0.769	0.769	0.769	0.769
		DS	0.949	0.947	0.864	0.949	0.864
		IPTW KM	0.949	0.951	0.949	0.915	0.915
		IPTW CH	0.948	0.949	0.948	0.915	0.915
		Matching	0.946	0.950	0.946	0.919	0.919
		EL	0.977	0.989	0.977	0.935	0.935
		AIPTW	0.953	0.953	0.952	0.950	0.920

Methods for deriving survival curves: KM, Standard Kaplan-Meier based approach (unadjusted); DS, Direct Standardization via a Cox model (G-formula); IPTW KM, Xie and Liu's approach; IPTW CH, Cole and Hernan's approach; Matching, Propensity score matching; EL, Empirical Likelihood approach; AIPTW, Augmented Inverse Probability of Treatment Weighting approach.

Case	Outcome model	Treatment model
1	Correct	Correct
2	Included extra variables, X_3 and X_6	Included extra variables, X_1 and X_4
3	Failed to include X_2	Correct
4	Correct	Failed to include X_2
5	Failed to include X_2	Failed to include X_2

Table C16: Median Length of 0.95 Confidence Interval for log(RAH)

			Case				
Scenario	Censoring	Method	1	2	3	4	5
1	C	KM	0.5823	0.5823	0.5823	0.5823	0.5823
		DS	0.3686	0.3877	0.3721	0.3686	0.3721
		IPTW KM	0.6845	0.6607	0.6845	0.6509	0.6509
		IPTW CH	0.6801	0.6557	0.6801	0.6480	0.6480
		Matching	0.7986	0.7954	0.7986	0.7537	0.7537
		EL	1.2438	1.8072	1.2438	0.7255	0.7255
		AIPTW	0.5690	0.5745	0.5704	0.5579	0.5478
2	C	KM	0.6558	0.6558	0.6558	0.6558	0.6558
		DS	0.4306	0.4543	0.4383	0.4306	0.4383
		IPTW KM	0.7526	0.7181	0.7526	0.7204	0.7204
		IPTW CH	0.7464	0.7113	0.7464	0.7159	0.7159
		Matching	0.8757	0.8732	0.8757	0.8352	0.8352
		EL	1.3282	1.9294	1.3282	0.8002	0.8002
		AIPTW	0.6187	0.6255	0.6220	0.6011	0.5973

Methods for deriving survival curves: KM, Standard Kaplan-Meier based approach (unadjusted); DS, Direct Standardization via a Cox model (G-formula); IPTW KM, Xie and Liu's approach; IPTW CH, Cole and Hernan's approach; Matching, Propensity score matching; EL, Empirical Likelihood approach; AIPTW, Augmented Inverse Probability of Treatment Weighting approach.

Case	Outcome model	Treatment model
1	Correct	Correct
2	Included extra variables, X_3 and X_6	Included extra variables, X_1 and X_4
3	Failed to include X_2	Correct
4	Correct	Failed to include X_2
5	Failed to include X_2	Failed to include X_2

D Impact of censoring rate on inference based on the Average Hazard approach and Cox's approach

In response to a reviewer's suggestion, we conducted a simulation study to examine how varying censoring rates influence inference for the RAH compared with Cox's hazard ratio. This numerical investigation was carried out in a simple two-sample setting without covariate adjustment, designed to sensitively capture the effect of censoring on the performance of Ratio of Average Hazard (RAH) and Cox's Hazard Ratio (HR).

D.1 Simulation Configurations

Survival times for the control arm (T_0) were generated from an exponential distribution with rate parameter $\lambda_0 = 0.2$, and survival times for the treatment arm (T_1) were generated from an exponential distribution with rate $\lambda_1 = \lambda_0 \times \mathrm{HR}$, where the true hazard ratio was set to HR = 0.8. Under this configuration, the RAH also equals 0.8, regardless of the choice of truncation time τ.

Independent censoring times (C^*) were generated from an exponential distribution with rate λ_C chosen such that $\Pr(C^* \leq 10)$ equaled a prespecified censoring proportion, ranging from 30% to 95%. The observed times were then defined as $\min(T_0, C^*, 10)$ and $\min(T_1, C^*, 10)$ for the control and treatment groups, respectively.

We generated 5,000 data sets for each sample size ($n = 200$ and $n = 500$). For each replicate, we estimated the HR using a Cox proportional hazards model and the RAH using the Kaplan-Meier plug-in method [12] with truncation at $\tau = 10$. Performance was evaluated in terms of: 1) bias of the point estimates, 2) coverage probabilities of the 95% confidence intervals, and 3) the average estimated standard errors, all assessed on the log scale for both HR and RAH.

It is important to note that the RAH cannot be estimated using the Kaplan-Meier plug-in method when the risk set at τ is empty in either the treatment or control group, a situation that may arise under heavy censoring or small sample sizes. In contrast, HR estimation based on Cox's model is not subject to this limitation. In the simulation, we recorded the number of replicates in which the risk set at τ was empty in either group and excluded those replicates when evaluating the performance of the nonparametric inference for RAH. Consequently, the reported results for the RAH may be based on fewer than 5,000 replicates, whereas the HR results always used the full set of 5,000 replicates.

D.2 Simulation Results

Cox's HR performed well regardless of the censoring rate, showing negligible bias and coverage probabilities close to the nominal 95% level. Except under heavy censoring, the RAH also yielded results comparable to those of Cox's HR (Table D1).

In particular, when censoring was 80%, 90%, or 95%, some simulation replicates had an empty risk set at τ in either the treatment or control group. As the frequency of such replicates increased, the RAH exhibited bias and the coverage probability deviated slightly from the nominal level. These issues did not arise for Cox's HR, for which all 5,000 replicates contributed to inference.

The poorer performance of the RAH under heavy censoring is attributable to biased sampling: replicates in which the RAH was not estimable had to be excluded, a notable limitation of the nonparametric estimation method. The results further indicated that the impact of censoring on the performance of the RAH strongly depends on sample size. With $n = 200$, performance degraded when the censoring rate reached 90% or higher, whereas with $n = 500$, performance remained satisfactory even at 90% censoring. Thus, the key design consideration is not merely the censoring

rate but the expected size of the risk set at τ. When designing a study, it is important to ensure that the risk set size at the chosen truncation time does not become too small.

Overall, these findings suggest that the RAH is generally robust, with performance comparable to Cox's HR in most settings, and that careful attention to maintaining an adequate risk set at τ can ensure reliable inference.

Table D1: Bias, coverage probability of 95% CI, and average standard error (Ave SE) comparing Cox's HR and RAH under varying censoring rates and sample size based on 5000 iterations.

		Log(HR)			Log(RAH)			
n	Censoring	Bias	Coverage	Ave SE	Excluded*	Bias	Coverage	Ave SE
200	0.30	0.000	0.956	0.117	0	-0.001	0.953	0.097
200	0.40	-0.001	0.952	0.120	0	-0.001	0.951	0.099
200	0.50	-0.001	0.953	0.124	0	-0.001	0.948	0.103
200	0.60	0.000	0.950	0.128	0	-0.001	0.947	0.107
200	0.70	0.000	0.950	0.134	0	0.000	0.947	0.112
200	0.80	0.000	0.956	0.141	16	-0.002	0.942	0.121
200	0.90	0.001	0.950	0.154	385	-0.007	0.940	0.138
200	0.95	0.002	0.949	0.166	1771	-0.018	0.936	0.159
500	0.30	0.002	0.952	0.074	0	0.002	0.949	0.066
500	0.40	0.002	0.950	0.076	0	0.002	0.950	0.068
500	0.50	0.002	0.951	0.078	0	0.002	0.950	0.070
500	0.60	0.002	0.948	0.081	0	0.002	0.947	0.073
500	0.70	0.002	0.948	0.084	0	0.002	0.947	0.077
500	0.80	0.002	0.946	0.089	0	0.003	0.945	0.083
500	0.90	0.001	0.949	0.097	5	0.002	0.942	0.096
500	0.95	0.001	0.947	0.104	207	-0.001	0.936	0.111

* For log(RAH), the column "Excluded" indicates the number of replicates (out of 5,000) in which nonparametric estimation for $AH(\tau)$ was not feasible due to an empty risk set at $\tau = 10$ in either group.

References

[1] Rosenbaum P, Rubin D. The Central Role of the Propensity Score in Observational Studies for Causal Effects. Biometrika. 1983 Apr;70(1):41-55.

[2] Ozenne B, Scheike T, Stærk L, Gerds T. On the estimation of average treatment effects with right-censored time to event outcome and competing risks. Biometrical Journal. 2020;62(3):751-63.

[3] Zhang M, Schaubel D. Contrasting treatment-specific survival using double-robust estimators. Statistics in medicine. 2012;31(30):4255-68.

[4] Chang I, Gelman R, Pagano M. Corrected group prognostic curves and summary statistics. Journal of chronic diseases. 1982;35(8):669-74.

[5] Makuch R. Adjusted survival curve estimation using covariates. Journal of chronic diseases. 1982;35(6):437-43.

[6] Robins J. A new approach to causal inference in mortality studies with a sustained exposure period—application to control of the healthy worker survivor effect. Mathematical modelling. 1986;7(9-12):1393-512.

[7] Xie J, Liu C. Adjusted Kaplan–Meier estimator and log-rank test with inverse probability of treatment weighting for survival data. Statistics in medicine. 2005;24(20):3089-110.

[8] Kaplan E, Meier P. Nonparametric estimation from incomplete observations. Journal of the American statistical association. 1958;53(282):457-81.

[9] Cole S, Hernán M. Adjusted survival curves with inverse probability weights. Computer methods and programs in biomedicine. 2004;75(1):45-9.

[10] Austin P. The use of propensity score methods with survival or time-to-event outcomes: reporting measures of effect similar to those used in randomized experiments. Statistics in medicine. 2014;33(7):1242-58.

[11] Wang X, Bai F, Pang H, George S. Bias-adjusted Kaplan–Meier survival curves for marginal treatment effect in observational studies. Journal of biopharmaceutical statistics. 2019;29(4):592-605.

[12] Uno H, Horiguchi M. Ratio and difference of average hazard with survival weight: New measures to quantify survival benefit of new therapy. Stat Med. 2023;42(7):936-52.
